# Influenza in Migratory Birds and Evidence of Limited Intercontinental Virus Exchange

**DOI:** 10.1371/journal.ppat.0030167

**Published:** 2007-11-09

**Authors:** Scott Krauss, Caroline A Obert, John Franks, David Walker, Kelly Jones, Patrick Seiler, Larry Niles, S. Paul Pryor, John C Obenauer, Clayton W Naeve, Linda Widjaja, Richard J Webby, Robert G Webster

**Affiliations:** 1 Division of Virology, Department of Infectious Diseases, St. Jude Children's Research Hospital, Memphis, Tennessee, United States of America; 2 Hartwell Center for Bioinformatics and Biotechnology, St. Jude Children's Research Hospital, Memphis, Tennessee, United States of America; 3 Conserve Wildlife Foundation, Bordentown, New Jersey, United States of America; 4 Environment Canada, Canadian Wildlife Service, Edmonton, Alberta, Canada; 5 Department of Structural Biology, St. Jude Children's Research Hospital, Memphis, Tennessee, United States of America; University of Wisconsin-Madison, United States of America

## Abstract

Migratory waterfowl of the world are the natural reservoirs of influenza viruses of all known subtypes. However, it is unknown whether these waterfowl perpetuate highly pathogenic (HP) H5 and H7 avian influenza viruses. Here we report influenza virus surveillance from 2001 to 2006 in wild ducks in Alberta, Canada, and in shorebirds and gulls at Delaware Bay (New Jersey), United States, and examine the frequency of exchange of influenza viruses between the Eurasian and American virus clades, or superfamilies. Influenza viruses belonging to each of the subtypes H1 through H13 and N1 through N9 were detected in these waterfowl, but H14 and H15 were not found. Viruses of the HP Asian H5N1 subtypes were not detected, and serologic studies in adult mallard ducks provided no evidence of their circulation. The recently described H16 subtype of influenza viruses was detected in American shorebirds and gulls but not in ducks. We also found an unusual cluster of H7N3 influenza viruses in shorebirds and gulls that was able to replicate well in chickens and kill chicken embryos. Genetic analysis of 6,767 avian influenza gene segments and 248 complete avian influenza viruses supported the notion that the exchange of entire influenza viruses between the Eurasian and American clades does not occur frequently. Overall, the available evidence does not support the perpetuation of HP H5N1 influenza in migratory birds and suggests that the introduction of HP Asian H5N1 to the Americas by migratory birds is likely to be a rare event.

## Author Summary

Influenza surveillance in wild migratory birds has been done at two sites in North America: 1) in Alberta, Canada, for the past 31 years, and 2) along Delaware Bay, United States, for the past 22 years. These studies support the concept that wild migratory birds are the reservoirs of all influenza A viruses and that the influenza viruses in the world can be divided into two distinct superfamilies, one in Eurasia and the other in the Americas. From time to time these viruses spread to domestic poultry and to humans and cause pandemics of disease. Many investigators have expanded these studies particularly in Europe, Asia, and the Americas. The emergence of highly pathogenic H5N1 in Asia a decade ago and the continuing evolution and spread of these H5N1 viruses to the whole of Eurasia is a continuing problem for veterinary and human public health. The available evidence from Eurasia is that migratory birds can be infected and may be involved in local spread of the highly pathogenic H5N1 virus. The question addressed in the present study is why the highly pathogenic H5N1 influenza virus has not yet reached the Americas despite the overlap in migratory bird pathways, particularly in Alaska. Genomic analysis of influenza viruses from our repository failed to provide evidence of influenza viruses with their whole genome originating from Eurasia. However, we found occasional influenza viruses from North America with single or multiple genes that originated in Eurasia. Our interpretation is that while influenza viruses do exchange between the two hemispheres, this is a rare occurrence. Regardless, enhanced surveillance should be continued in the Americas in case this rare event occurs.

## Introduction

Long-term surveillance of influenza in migratory waterfowl in North America [1–4] from 1976 to the present and more intensive surveillance in Europe from 1998 to the present [5,6] have established the importance of Anseriformes (waterfowl) and Charadriiformes (gull and shorebird) in the perpetuation of all known subtypes of influenza A viruses. The available evidence suggests that each of the 16 hemagglutinin (HA) and nine neuraminidase (NA) subtype combinations exist in harmony with their natural hosts, cause no overt disease, and are shed predominantly in the feces. Although highly pathogenic (HP) influenza viruses have occasionally been isolated from wild migratory birds, including H5N3 A/Tern/South Africa/61 [7] and H7N1 from an outbreak of HP avian influenza in Italy in 1999–2000 [8], the usual finding is that each of the HP H5 and H7 lineages that emerge in gallinaceous poultry originate from different nonpathogenic precursors—e.g., A/Chicken/Pennsylvania/83 (H5N2) [9], A/Chicken/Mexico/94 (H5N2) [10], A/Chicken/Netherlands/2003 (H7N7) [11], and A/Chicken/Canada/2004 (H7N3) [12].

There has been overall agreement between the findings of various influenza surveillance studies in migratory birds in regard to the role the birds play in the emergence of pandemics in humans, lower animals, and domestic poultry. The only significant difference between the findings for influenza surveillance in aquatic birds in the Americas and in Europe is the role of shorebirds; in Europe, influenza viruses to date have rarely been isolated from shorebirds, while in the Americas, the available evidence supports the notion that shorebirds carry influenza viruses during their migration from South America to their North American breeding grounds each May. Our earlier studies also showed that shorebirds and gulls in the Americas are more frequently the source of the potential precursors to HP H5 and H7 avian influenza viruses [3], while in Eurasia, the precursors of HP influenza viruses are usually from duck species [13,14].

An important unanswered question is how often the influenza viruses in wild migratory birds in Eurasia spread to the Americas and establish lineages on that continent, and vice versa. This is currently a question of great concern to both veterinary and public health officials in the Americas. The continuing circulation of the Asian HP H5N1 in several countries in Eurasia and the reemergence in the winter of 2006–2007 of HP H5N1 in South Korea, Japan, Thailand, Vietnam, and China supports the contention that this H5N1 virus is being perpetuated in this region. One of the many unanswered questions is whether the HP avian H5N1 virus is being perpetuated in domestic poultry or in wild bird species.

Examination of the global migratory pathways of migratory waterfowl shows overlaps between eastern Eurasia and Alaska and between Europe and eastern North America [15]. This leads to the question of why the HP H5N1 viruses have not arrived in the Americas. Phylogenetic analysis of influenza viruses from migrating birds generally divides them into two large polyphyletic clades or superfamilies: one in Eurasia, the other in the Americas [6,16–18]. Whereas most of the known subtypes of influenza A viruses have been detected in each hemisphere, the rarely isolated H14 and H15 subtypes have, to date, been detected only in Eurasia. While influenza viruses or gene segments have been shown to exchange between parts of Eurasia and the Americas [19–24], the frequency of exchange of all eight gene segments of influenza viruses is unknown. However, based on the clear phylogenetic separation into two superfamilies [25], it might be predicted that the exchange of whole influenza viruses would be infrequent. Here, we utilize the genomic information from 248 complete influenza virus sequences and 6,767 gene segments [26] to estimate how often this occurs.

In our studies of influenza surveillance in wild ducks in Alberta, Canada, and in shorebirds and gulls at Delaware Bay (New Jersey), United States, between 2001 and 2006, we confirmed the presence of the H16 subtype in the Americas and the presence of a high frequency of infection of H7N3 viruses from shorebirds and gulls. We continued to detect H1 through H13 HA subtypes and N1 through N9 subtypes of NA but not H14 or H15 in ducks, shorebirds, or gulls. We found no virologic evidence of the HP H5N1 virus in ducks, shorebirds, or gulls or serologic evidence in ducks sampled in Alberta. Our genomic analysis of the full sequence of 248 influenza genomes showed no viruses whose entire genome transferred between the two hemispheres. The size of our complete-genome virus data set, however, may be too small to detect a transferred virus before it had opportunities to reassort.

## Results

### Virus Isolation and Prevalence

**Figure 1:**
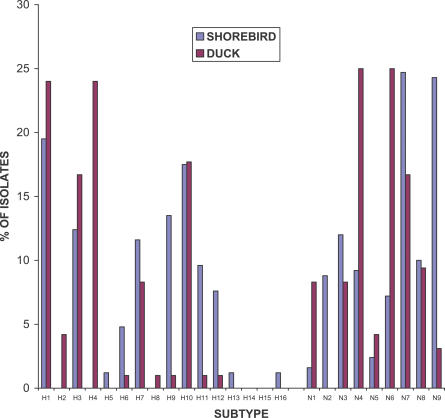
Comparison of the Frequencies of Influenza A Virus HA and NA Subtypes from Wild Ducks in Alberta, Canada, and Shorebirds in Delaware Bay (New Jersey), United States Frequency was determined for each group (i.e., wild ducks or shorebirds) as a percentage of the total number of positive isolates of a subtype per total number of positive samples for that group.

The rapid spread of HP H5N1 influenza from Qinghai Lake, China, to Europe and Africa raised the possibility that migratory birds were involved [15]. The reemergence of HP H5N1 influenza virus in domestic poultry in Japan, South Korea, and Thailand in January 2007 after successful eradication of these viruses in 2003 and 2004 raised the question of the mode of spread and raised the level of concern for further spread of these viruses. To determine the prevalence of different subtypes of influenza A viruses at two sites in North America, virologic surveillance was done in wild ducks in Alberta and in shorebirds and gulls at Delaware Bay from 2001 through 2006. Prospective surveillance has been done for the past 30 years in wild ducks in Alberta and for 21 years in shorebirds and gulls at Delaware Bay. During the five years of our study, 590 cloacal samples from wild ducks yielded 98 influenza A viruses (isolation rate, 16.6%), while 1970 fecal samples from shorebirds and gulls yielded 114 influenza A isolates (isolation rate, 5.8%). Antigenic analysis of the influenza virus isolates from ducks, shorebirds, and gulls established the continuing circulation of HA subtypes H1 through H13 and NA subtypes N1 through N9 (Figure 1). We also report the isolation of the newly characterized H16 subtype in shorebirds. Subtypes H2, H4, and H8 were identified only in ducks, while subtypes H5, H13, H16, and N2 were recovered only from shorebirds and gulls.

Subtypes that occurred in both ducks and shorebirds, but with much higher prevalence in shorebirds, were H9, H11, H12, and N9. In contrast, although they were recovered from both ducks and shorebirds, N1, N4, and N6 were more prevalent in ducks.

Five HA subtypes accounted for 75% of the isolates found in shorebirds: H1 (19.5%), H10 (17.5%), H9 (13.5%), H3 (12.4%), and H7 (11.6%), whereas four HA subtypes represented 82% of the viruses identified in ducks: H1 (24.0%), H4 (24.0%), H10 (17.7%), and H3 (16.7%). For the NA subtypes in shorebirds, the N7 (24.7%) and N9 (24.3%) isolates were dominant, and for ducks, N4 (25.0%) and N6 (25.0%) were the most prevalent subtypes. We isolated only three H5 viruses, all of which came from shorebirds in 2004 and were paired with either N7 or N8. We identified a total of 49 HA and NA subtypes (out of a possible 144), with 25 subtypes being found in ducks and 31 subtypes being found in shorebirds. There were 17 subtypes that were isolated only from ducks and 24 subtypes that were recovered only from shorebirds. The most highly represented subtypes found in ducks were H4N6 (21.9%), H1N4 (18.8%), and H10N7 (16.7%), and the most numerous subtypes identified in shorebirds were H10N7 (17.5%), H1N9 (15.1%), and H7N3 (19.8%). No H5N1 was detected during the study period.

The isolation of high numbers of H7N3 influenza viruses from gulls and shorebirds in May 2006 was different from the results of previous years. These H7N3 viruses are examined in more detail below.

### Serologic Surveillance

To determine the prevalence of infection of wild ducks with the influenza viruses that are considered more likely to cause a pandemic in domestic fowl or in humans, hemagglutination inhibition (HI) studies were done with H5, H6, H7, and H9 influenza viruses (Table 1). The influenza viruses chosen for use were those that were of current relevance in Asia and the Americas. Low levels of HI antibodies to each of the viruses tested were detected each year with an overall higher rate of detection of H9 followed by H5, H6, and H7 antibodies. Most positive sera reacted with HI titers of 1/10 to 1/20, indicating that the birds were probably infected with a virus of the homologous subtype but probably not with viruses identical to those used in the test. Studies done yearly from 2004 through 2006 on adult ducks showed no trend toward higher numbers of birds infected with H5, H6, H7, or H9. Thus, serologic studies confirmed the continued circulation of each of the influenza subtypes.

**Table 1:**
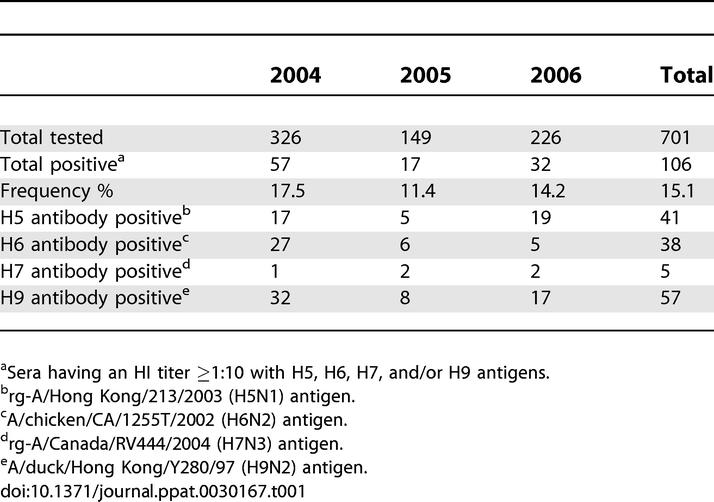
Serologic Surveillance for Influenza Virus Infection of Migratory Ducks, Alberta, Canada

### The H16 Influenza Viruses from North America

A new subtype of influenza A viruses from black-headed gulls in Sweden was recently characterized and designated A/Black-headed gull/Sweden/2/99 (H16N3) [27]. Large-scale sequence analysis of avian influenza viruses [26] established that viruses of the H16 subtype are present in the American superfamily. The first of these viruses, A/Black-legged Kittiwake/Alaska/295/75 (H16N3), was isolated in 1975, and later isolates were detected in shorebirds in 1986 and in herring gulls in 1988.

**Figure 2:**
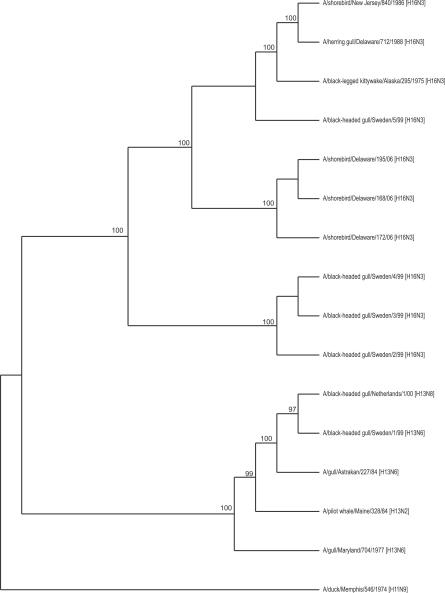
Phylogenetic Tree for the HA Gene (Nucleotides 35–1729) of H13 and H16 Influenza A Viruses The nucleotide sequences were analyzed by PHYLIP 3.66 software [41] using the neighbor-joining method with 100 bootstraps. The tree was rooted with the A/Duck/Memphis/546/74 (H11) HA sequence.

In our study, three H16N3 influenza viruses (A/Shorebird/DE/168/06, A/Shorebird/DE/172/06, and A/Shorebird/DE/195/06) were isolated from shorebirds. No H16 viruses were detected in samples from duck species. Phylogenetic analysis showed that the HA of these viruses could be divided into two subgroups, one from Europe and the other from North America (Figure 2). It is noteworthy that A/Black-headed gull/Sweden/5/99 (H16N3) grouped with the North American H16 isolates, suggesting a possible transfer between North America and Europe. However, it is not possible to determine if the HA gene originated in North America or Europe.

Antigenic analysis of the H16 influenza virus isolates from North America with postinfection ferret sera (Table 2) showed that the viruses were antigenically distinguishable; two of the current H16 isolates (SB/DE/172/06 and SB/DE/195/06) were indistinguishable from each other, but the SB/DE/168/06 and BHG/Sweden/5/99 reacted to 4-fold lower HI titers (1/80 versus 1/320). The H16N3 isolate from 1975 also reacted to a titer of 1/80, while the 1986 and 1988 viruses reacted to a titer of 1/40. Thus, the H16 viruses showed antigenic and genetic diversity in their natural hosts.

**Table 2:**
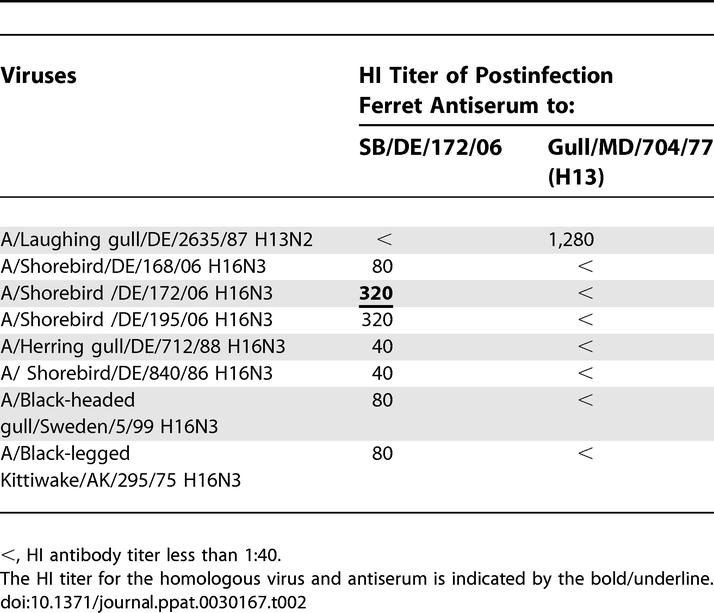
Antigenic Analysis of H16N3 Influenza Viruses with Ferret Antisera

### The H7N3 Viruses

Two H7N3 influenza viruses of the American superfamily of influenza viruses have in the recent past evolved into HP viruses. One of these HP H7N3 viruses occurred in Chile, South America, A/Chicken/Chile/4322/02 (H7N3) [28]; the other was in British Columbia, Canada, A/Chicken/British Columbia/NS-2035–12/04 (H7N3) [12]. Each of these viruses evolved from nonpathogenic H7N3 precursor viruses, and the available evidence indicates they acquired the HP characteristic by recombination; they inserted additional amino acids at the cleavage site of the HA [29,30] from one of their other gene segments.

In 2006, we isolated 24 H7N3, one H7N4, and one H7N5 influenza viruses from shorebirds and gulls at Delaware Bay. Each of these viruses was passaged once in chicken eggs after the initial egg isolation, produced high HA titers (1:640–1:4,096), and 20 of the viruses killed the chicken embryos by 48 h after injection. To determine whether these viruses could also cause death of chickens, the A/Laughing gull/DE/42/06 (H7N3) isolate was inoculated into four young adult white leghorn chickens intranasally, intratracheally, and intraocularly with 10^8.75^ egg infectious doses_50_ (EID_50_). The birds showed no loss of appetite or disease signs; virus was reisolated from tracheal or cloacal samples of three of the four chickens (results not shown). The H7N3 isolate induced high levels of HI antibodies in the inoculated chickens. In HI tests using postinfection antiserum, the 26 H7 viruses were antigenically homogeneous with HI titers mostly within 2-fold of each other (1:160, 1:320, or 1:640). To further evaluate the pathogenic potential of the viruses, two representative H7N3 isolates (A/Shorebird/DE/22/06 and A/Laughing gull/DE/42/06) were inoculated intravenously with 10^8.5^ and 10^8.75^ EID_50_, respectively, into ten chickens each to determine their intravenous pathogenicity index (IVPI). Virus was detected from the cloaca of all the birds on day 3 postinfection. Although neither of the H7N3 viruses tested were HP, one bird did die 7 d after inoculation with A/Laughing gull/DE/42/06, and four of the birds were reported as lethargic with an IVPI of 0.28. Tissue samples from the dead bird had modest levels of virus in the pancreas (10^2.5^ EID_50_/ml) and the highest levels in the kidney (10^5^ EID_50_/ml). No virus was detected in samples from the brain, lung, heart, liver, or spleen of the dead bird. Repassaging of the virus from the kidney into ten chickens intravenously did not kill any of the birds. To determine whether the virus isolated from the dead chicken was capable of replicating in the absence of exogenous trypsin, a characteristic of HP viruses, we performed a plaque assay in MDCK cells with and without trypsin. Plaques were produced only in the presence of trypsin, indicating that the virus reisolated from the chicken could not be characterized as possessing this trait of HP influenza viruses.

**Figure 3:**
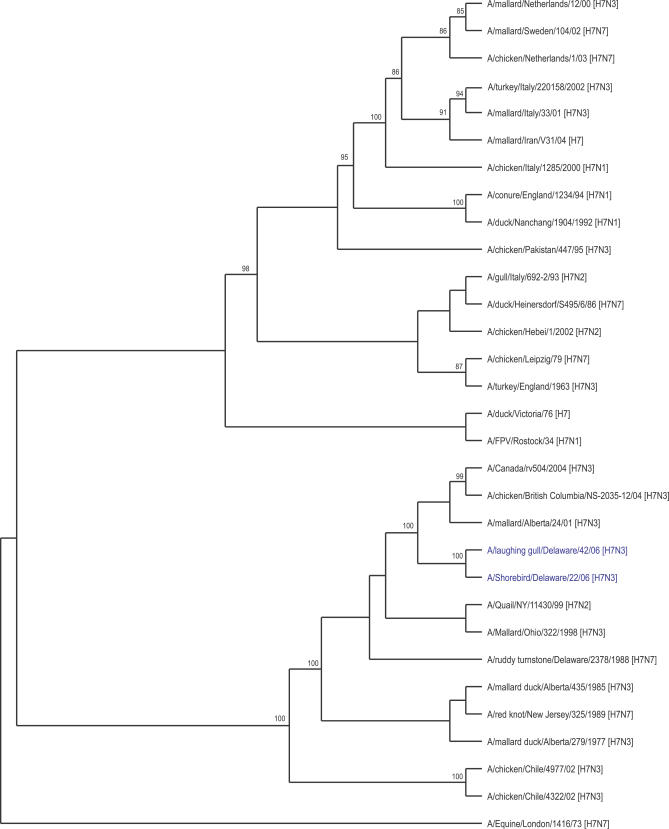
Phylogenetic Tree for the HA-1 Gene (Nucleotides 76–1026) of H7 Influenza A Viruses The nucleotide sequences were analyzed by PHYLIP software, version 3.66 [41], using the neighbor-joining method with 100 bootstraps. The tree was rooted with the A/Equine/London/1416/73 HA sequence.

Phylogenetic analysis of the HA of the H7N3 viruses from gulls and shorebirds at Delaware Bay in 2006 shows they reside on a neighboring branch to that of the HP H7N3 viruses from chickens in British Columbia in 2004 (blue text, Figure 3). Although the viruses were related to those found in British Columbia, the connecting peptide of the HA of the shorebird viruses had the typical sequence of a nonpathogenic H7 virus. Phylogenetic analysis of the internal genes and the NA gene showed that all genes reside in the American clade, and the PB2, PB1, NP, NA, M, and NS genes are located on a branch adjacent to the HP H7N3 viruses from British Columbia (Figure S1).

A BLAST search was performed to determine the similarity of the shorebird and gull H7N3 viruses with the HP strains isolated in British Columbia. Nucleotide identity was high—93.8% to 98.9%—for all genes except PA, which had an identity of 87.8%.

Even though these H7N3 viruses are classified as nonpathogenic, they clearly have some potential for replication in domestic chickens and have the unusual characteristic of killing chicken embryos.

### Exchange of Influenza Genes and Viruses between the Major Continents

The detection of the HA of the H16 Swedish black-headed gull influenza virus among the rarely identified H16 viruses from North America (Figure 2) raised the question of the frequency of influenza virus transfer between continents. To investigate this, we examined the 6,767 genome segments of influenza viruses from wild birds, described previously by Obenauer et al. [26] (Table 3; Figures S2–S9). These viruses were generally separable into two large polyphyletic clades, one made up of viruses from the Americas, the other composed of viruses from Eurasia (and including Australia and Africa). In addition, we found a small number of viral segments isolated in either the Americas or Eurasia that clustered with strains from the opposing hemisphere (Table S1). Any remote segment or paraphyletic clade of segments, which clustered in the above manner, was termed as an outsider event.

**Table 3:**
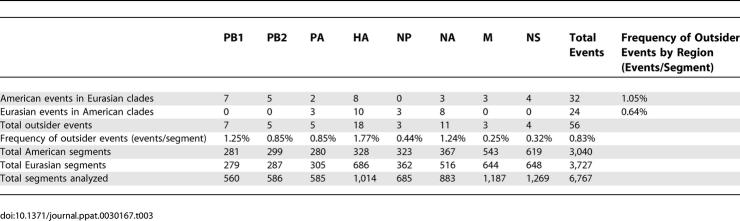
Frequency of Detection of Outsider Events

When the 6,767 influenza A gene segments were examined, 3,040 were found have been isolated in the Americas, while 3,727 were isolated in Eurasia (including Australia and Africa) (Table 3). Among the 3,040 American segments, there were 32 total outsider events, encompassing 101 total segments (Table S1), of which 30 were from Anseriformes (ducks) and 71 were from Ciconiiformes (which for our phylogenetic analyses included Charadriiforms [shorebirds and gulls]) isolated in Eurasia. Among the 3,727 Eurasian clade segments, there were 24 outsider events, encompassing 35 total segments, of which 20 were from Anseriformes and 15 from Ciconiiformes isolated in the Americas. Among the internal segments (PB1, PB2, PA, NP, M, and NS) no outsiders were present in all six segments (Table S1), indicating that, among the viruses examined, no entire viral genome from one clade was detected in the other clade. However, the 24 outsider events among the internal gene segments were detected in a non-linked fashion. Outsider events involving American isolated strains occurred most often with HA and PB1 genes (eight and seven events, respectively). Fewer events involving American strains were found with the remaining segments, ranging from five events (PB2) to zero events (NP). In contrast to the American outsider events found in Eurasian clades, the detection of outsider Eurasian events in American clades was lower. Eurasian virus outsider genes were only detected for HA (ten events), NA (eight events), PA (three events) and NP (three events). The overall more frequent detection of the HA and NA outsider events probably reflects the ability of these genes to exchange by reassortment and to be maintained in the population, presumably after the rare occurrence of an exchange of entire viruses between the continents. Thus, there does not appear to be a restriction on compatibility of the HA and NA genes between the two superfamilies. When the internal genes were compared, PB2, PB1, M, and NS showed marked differences in the number of outsider segments detected in American clades compared to the number of outsider segments found in Eurasian clades. None of these segments were identified as outsiders in the American clades; however, the Eurasian clades had seven PB1 American wild bird outsiders while PB2 had five, M had three, and NS had four.

When the two types of aquatic birds were compared, the Ciconiiformes (shorebirds and gulls) more frequently contained outsider gene segments than the Anseriformes (ducks). Among all the Ciconiiformes (*n* = 728)*,* 9.8% of American virus gene segments were detected in Eurasian clades, and 2.1% of Eurasian virus gene segments resided in American clades. The frequency of outsider segments among all Anseriformes (*n* = 3,436), however, was similar across both hemispheres: 0.87% of the outsider segments were isolated in the Americas, whereas 0.58% were isolated in Eurasia. These figures represent the relative frequency of detection of outsider gene segments in either the Eurasian or American clades, but they do not give a measure of the frequency of exchange of the entire virus (all eight segments) between continents. In our studies, we failed to detect any complete genome exchanges among the 248 viruses examined (Table S2). Thus, the frequency of exchange was less than 0.6%.

## Discussion

Virologic surveillance of apparently healthy birds has established that the waterfowl of the world are the natural reservoirs of all known influenza A viruses. The Anseriformes (waterfowl) and Charadriiformes (shorebirds and gulls) are the major reservoirs in which the 16 HA and nine NA subtypes are perpetuated. However, a wide range of birds can support limited replication but do not perpetuate influenza A viruses.

In this study, we characterized the H16 influenza viruses from the Americas that were first described in Europe in 2004 [27] and found an unusually high frequency of H7N3 influenza viruses from shorebirds and gulls at Delaware Bay in 2006. Despite virologic and serologic surveillance in migratory waterfowl, no evidence of the Asian HP H5N1 influenza virus was found. Continued prospective surveillance at two sites—one in Alberta for ducks and another at Delaware Bay for shorebirds and gulls—established the continued circulation of HA subtypes H1 through H13 and H16 and all nine NA subtypes. However, the rare H14 and H15 subtypes were not detected.

One frequently mentioned possibility is that wild migratory birds from Eurasia will carry the Asian HP H5N1 to the Americas. There are several articles describing the detection of influenza viruses belonging to Eurasian phylogenetic clades in the Americas and vice versa [19–24]. Each of these studies has been based on analysis of single genes (e.g., the HA, M, etc.). To date, we know of no study that has investigated the spread between the continents of entire influenza genomes containing all eight segments. In this study, we examined 6,767 individual gene segments and 248 entire influenza genomes. Phylogenetic analysis supported the contention that the influenza viruses are clearly separable into distinct Eurasian and American superfamilies [6,16–18,20]. We measured the frequencies of gene segments belonging to one superfamily occurring among influenza virus isolates in the other superfamily, defined here as outsider events, and found low frequencies of inter-hemispheric gene transfer. The rates varied according to gene segment, ranging from 0.25% for M (three outsider events seen among 1,187 sequences) to 1.77% for HA (18 events among 1,014 sequences). Our analysis of entire influenza genomes containing all eight gene segments revealed no detectable whole-genome transfers between superfamilies among the 248 viruses examined. After such an event occurs, there are relatively frequent reassortment events involving the surface glycoprotein genes (HA and NA), implying that there is no restriction in compatibility between the internal genes of one superfamily and the surface glycoproteins of the other superfamily. The internal genes are exchanged at a lower frequency and maintained, but there are no obvious patterns implying linkage of genes. Based on these studies, it is more likely that the Asian H5N1 viruses will be imported into the Americas with birds moved legally or illegally by humans. The available evidence is that at least one of the introductions of Asian HP H5N1 into Nigeria was with poultry that was imported either alive or frozen [31].

The recent introduction of HP H5N2 of American lineage into Japan indicates another mechanism for moving influenza virus between continents. The close genetic similarity between the HP H5N2 and the vaccine strain of H5N2 used in poultry in Central America suggest that this virus was transferred with smuggled vaccine [32]. Japan has a strict ban on the use of agricultural vaccines, yet the HP H5N2 of American lineage was introduced into Japan and caused lethal disease in poultry. Whether this originated from improperly inactivated vaccine or from contaminated vaccine is unknown.

The earliest H16 in the repository at St. Jude Children's Research Hospital is A/Kittiwake/Alaska/295/75 (H16N3), indicating that this virus has been in the Americas for a considerable period. It is notable that an H16 virus from a black-headed gull in Sweden is included in the American phylogenetic lineage, indicating transfer of the HA between continents. To date, the H16 viruses have been isolated only from gulls and shorebirds but not from ducks. The H16 viruses have shown limited antigenic drift from 1975 to 2006 and, to date, have not been associated with disease in any species.

The detection of a cluster of H7N3 influenza viruses in shorebirds in May 2006 was not found in previous years and raises questions about the propensity of these H7N3 viruses to replicate well in gallinaceous poultry and kill chicken embryos. In experiments using four H7N3 influenza viruses isolated from shorebirds and wild ducks between 1977 and 2000, little or no replication of virus was observed in experimentally infected chickens. In contrast, most of the H7N3 viruses used in our study replicated to high titers after oral inoculation in chickens, and 20 of the 24 viruses killed chicken embryos.

Phylogenetic analyses and sequence homologies of the genome of this cluster of H7N3 isolates showed that these viruses are closely related to the H7N3 influenza strain isolated from poultry in British Columbia in 2004. Taken together, the data indicate the potential of these H7N3 viruses to replicate in domestic chickens and underline the importance of biosecurity in commercial poultry raising and the need to keep such influenza viruses out of commercial poultry so they do not have the opportunity to evolve into HP strains.

The continued reemergence of HP H5N1 influenza viruses from the hypothetical epicenter in Guangdong Province, China [33,34], over the past ten years raises the question of whether the HP Asian H5N1 viruses are being perpetuated in wild birds. Surveillance in migratory waterfowl does show that many species will support the replication of the HP H5N1 viruses [35]; however, to date there is no evidence that the Asian HP H5N1 viruses are being perpetuated in migratory waterfowl. Although the spread of HP H5N1 viruses from Qinghai Lake, China, to central Asia and Europe has been attributed to migratory birds [15], there is no evidence that the HP H5N1 viruses are being perpetuated in migratory waterfowl.

Although the number of samples collected per year from migratory ducks in Alberta and shorebirds and gulls at Delaware Bay are rather modest, they do support the findings of the recent extensive surveillance done by the Canadian Cooperative Wildlife Health Center [36] and by the United States Geological Survey [37]. Surveillance studies in more than 100,000 wild birds have provided support for the contentions that (1) waterfowl are a major reservoir of influenza viruses; (2) low-pathogenic H5 influenza viruses are present in these wild birds; and (3) the HP H7 viruses previously found in Canada were not detected. Similarly, studies done over the 30 years of influenza virus surveillance in wild birds [3] in the Americas found no evidence for the perpetuation of HP H5 or H7 influenza viruses in migratory birds.

The available evidence suggests that the perpetuation of HP H5N1 Asian influenza viruses occurs through domestic waterfowl [33,38]. In the cooler months of the year, the virus load in the infected but apparently healthy domestic waterfowl spills over into both migratory waterfowl and gallinaceous poultry flocks and reignites the spread of HP H5N1 viruses. There is still a paucity of surveillance data from migratory birds in Asia, but the extensive surveillance of migratory waterfowl in Europe [6] does not support the hypothesis that wild migratory birds are perpetuating the Asian HP H5N1 virus. Thus, the above studies on the low frequency of exchange of entire influenza virus genomes of nonpathogenic influenza viruses between Eurasia and the Americas, plus the absence of evidence of HP H5N1 virus perpetuation in the influenza viruses found in migratory waterfowl, make the probability of introduction of HP H5N1 into the Americas by migratory birds an unlikely event.

## Materials and Methods

### Sample collection and viruses.

During 2001–2006, influenza surveillance in wild ducks that began in Alberta in 1976 was continued. During the same years, surveillance studies that were initiated in 1985 in shorebirds migrating through Delaware Bay were continued annually. Collection sites, collection of specimens, virus isolation, and characterization of the isolates have been described previously [3,4]. The A/Black-headed gull/Sweden/5/99 H16N3 virus used in this study was kindly provided by A. D. M. E. Osterhaus, Erasmus Medical Center, Rotterdam, The Netherlands.

### Duck serology.

Healthy adult wild ducks, captured in Alberta by the Canadian Wildlife Service for banding and tracking migration, were bled in the field to obtain 3–4 ml of blood from each duck and were released. Blood was clotted in serologic tubes overnight at room temperature to separate the serum, and the serum was transferred by pipette to cryovials and stored in liquid nitrogen for shipment. In 2004, 326 serum samples were collected; in 2005, 149 serum samples were collected; and in 2006, 226 serum samples were collected. All sera were treated with receptor destroying enzyme overnight at 37 °C, inactivated at 56 °C for 30 min, and diluted 1:10 before antigenic testing. Sera were screened by HI test as previously described by Palmer [39] for antibodies to four subtypes of influenza antigen: H5N1, H6N2, H7N1, and H9N2. The representative viruses for each respective subtype were as follows: rg-A/Hong Kong/213/2003 (H5N1), A/Chicken/CA/1255T/2002 (H6N2), rg-A/Canada/RV444/2004 (H7N1), and A/Duck/Hong Kong/Y280/1997 (H9N2).

### Phylogenetic analysis.

*H7N3 and H16N3 phylogenies.* Gene sequences used for construction of phylogenetic trees were either obtained from the Los Alamos Influenza Sequence Database or from samples used in this study. The sequences were then aligned, and the ends were trimmed to equal lengths using BioEdit sequence alignment editor software, version 7.0.5 [40]. The neighbor-joining algorithm was applied using PHYLIP software, version 3.66 [41] and 100 bootstrap replicates. The H16N3 tree was rooted using an HA segment isolated from a distantly related H11 strain as an outgroup, whereas the H7N3 tree was rooted using an HA segment isolated from an equine host as its outgroup. The final tree outfile was visualized using TreeView Win32 software [42].

Outsider analyses. Two sets of phylogenetic trees were constructed using previously described methods [26] to infer the relationships among individual avian flu gene segments. The initial set analyzed a total of 6,767 segments (560 PB1, 586 PB2, 585 PA, 1014 HA, 685 NP, 883 NA, 1187 M, and 1267 NS) that were deposited in GenBank through April 2006 (Figures S2–S9). The second set of trees included 248 complete flu genomes, deposited into GenBank through February 2007 (Figures S10–S17). Any segment that was subsequently determined to be a duplicate sequence (as determined by strain name [Table S3]), isolated from domesticated fowl (Galliformes) or obtained from a non-avian source (e.g., blow fly or human) was excluded from further analyses. An outsider event was determined to have occurred if a strain isolated in one hemisphere was found to be clustered with a majority of strains from the opposing hemisphere. In the event that several closely related strains formed a clade within the strains from the opposing hemisphere, a single outsider event was determined to have occurred (Table S4). The 136 segments involved in the 56 outsider events, found in this study, represent 100 viral strains (Table S1). Only 68 of those strains represent cases where only a single segment underwent viral transfer; all of the segments, however, were considered for outsider analyses. Two large polyphyletic clades, containing either isolates from the Western Hemisphere or isolates from the Eastern Hemisphere, were generally identified for each gene segment. The exception to this was with HA and NA genes. Within both of these genes there are distinct amino acid sequences, which result in phylogenetically distinct subtypes. Since there were several cases in which certain subtypes of these genes were infrequently sequenced, it was decided that individual subtypes needed to be examined to avoid terming these infrequent sequences as outsider events (Table S5). In addition to grouping outsider events by gene segment, strains were additionally grouped by bird order. Eight known orders of wild birds were identified based on virus name, with unidentifiable birds grouped as “undetermined.” Because the orders Anseriformes and Ciconiiformes represent the source of the samples collected for virus isolation in this study, we selected those orders for more detailed analysis.

## Supporting Information

Figure S1 Phylogenetic Trees Used in the Analysis of the PB2, PB1, PA, NP, NA, M, and NS Genes of the H7N3 Viruses A/Shorebird/DE/22/2006 and A/Laughing Gull/DE/42/2006 The nucleotides used for the analysis are as follows: PB2, 69–2289; PB1, 25–2257; PA, 29–2151; NP, 46–1517; NA, 40–1444; M, 53–976; and NS, 41–859.
(67 KB PDF)

Figure S2 PB2 Phylogenetic Tree Used in the Outsider Analysis of 6,767 Avian Influenza A Virus Gene Segments (7.7 MB PDF)

Figure S3 PB1 Phylogenetic Tree Used in the Outsider Analysis of 6,767 Avian Influenza A Virus Gene Segments (7.4 MB PDF)

Figure S4 PA Phylogenetic Tree Used in the Outsider Analysis of 6,767 Avian Influenza A Virus Gene Segments (7.8 MB PDF)

Figure S5 HA Phylogenetic Tree Used in the Outsider Analysis of 6,767 Avian Influenza A Virus Gene Segments (3.6 MB PDF)

Figure S6 NP Phylogenetic Tree Used in the Outsider Analysis of 6,767 Avian Influenza A Virus Gene Segments (9.2 MB PDF)

Figure S7 NA Phylogenetic Tree Used in the Outsider Analysis of 6,767 Avian Influenza A Virus Gene Segments (11.9 MB PDF)

Figure S8 M Phylogenetic Tree Used in the Outsider Analysis of 6,767 Avian Influenza A Virus Gene Segments (3.3 MB PDF)

Figure S9 NS Phylogenetic Tree Used in the Outsider Analysis of 6,767 Avian Influenza A Virus Gene Segments (3.2 MB PDF)

Figure S10 PB2 Phylogenetic Tree Used in the Outsider Analysis of 248 Complete Avian Influenza A Virus Genomes (3.2 MB PDF)

Figure S11 PB1 Phylogenetic Tree Used in the Outsider Analysis of 248 Complete Avian Influenza A Virus Genomes (3.2 MB PDF)

Figure S12 PA Phylogenetic Tree Used in the Outsider Analysis of 248 Complete Avian Influenza A Virus Genomes (3.2 MB PDF)

Figure S13 HA Phylogenetic Tree Used in the Outsider Analysis of 248 Complete Avian Influenza A Virus Genomes (3.6 MB PDF)

Figure S14 NP Phylogenetic Tree Used in the Outsider Analysis of 248 Complete Avian Influenza A Virus Genomes (3.3 MB PDF)

Figure S15 NA Phylogenetic Tree Used in the Outsider Analysis of 248 Complete Avian Influenza A Virus Genomes (3.5 MB PDF)

Figure S16 M Phylogenetic Tree Used in the Outsider Analysis of 248 Complete Avian Influenza A Virus Genomes (3.3 MB PDF)

Figure S17 NS Phylogenetic Tree Used in the Outsider Analysis of 248 Complete Avian Influenza A Virus Genomes (3.2 MB PDF)

Table S1 Summary of Outsider Analysis of 6,767 Gene Segments (28 KB XLS)

Table S2 Summary of Outsider Analysis of 248 Complete Genomes (29 KB XLS)

Table S3 List of Duplicate Sequences Excluded from the Outsider Analysis (30 KB XLS)

Table S4 Summary of Outsider Events by Gene Segment (39 KB XLS)

Table S5 Frequency of Outsider Events in HA and NA Subtypes (14 KB XLS)

Table S6 GenBank Accession Numbers of Sequences Not Generated in this Study and Used for Phylogenetic Analysis of H7N3 Viruses (All Genes) and H16N3 Viruses (HA Gene) (115 KB XLS)

### Accession Numbers

Accession numbers for gene sequences produced by this study and deposited in GenBank (http://www.ncbi.nlm.nih.gov/Genbank/index.html) are EU030965–EU030988. The accession numbers of gene sequences that were not generated by this study, but were used for the analysis of the complete genome of the H7N3 viruses and the HA gene of the H16N3 viruses, can be found in Table S6.

## Abbreviations

HA: hemagglutinin
HI: hemagglutination inhibition
HP: highly pathogenic
NA: neuraminidase
