# Supplementary material for: Influenza in Migratory Birds and Evidence of Limited Intercontinental Virus Exchange
Source: PLoS Pathog. 2007 Nov 9;3(11):e167. doi: 10.1371/journal.ppat.0030167 (PMC2065878; doi:10.1371/journal.ppat.0030167)
Supplement: Supplementary file 1 [file ppat.0030167.sg001.pdf]

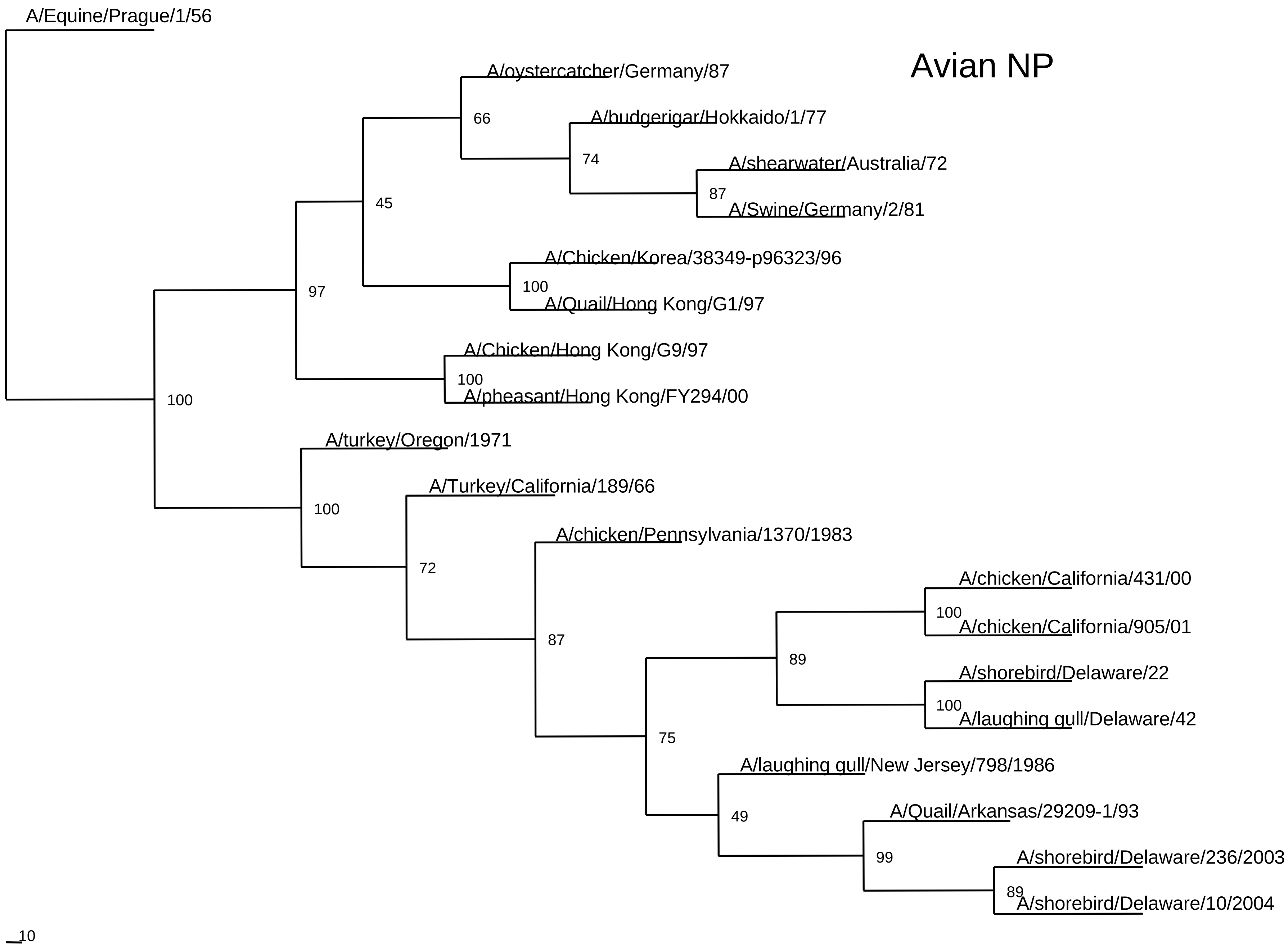
Avian NP
A/Equine/Prague/1/56
A/oystercatcher/Germany/87
66
A/budgerigar/Hokkaido/1/77
74
A/shearwater/Australia/72
87
A/Swine/Germany/2/81
45
A/Chicken/Korea/38349-p96323/96
100
A/Quail/Hong Kong/G1/97
97
A/Chicken/Hong Kong/G9/97
100
A/pheasant/Hong Kong/FY294/00
100
A/turkey/Oregon/1971
100
A/Turkey/California/189/66
72
A/chicken/Pennsylvania/1370/1983
87
A/chicken/California/431/00
100
A/chicken/California/905/01
89
A/shorebird/Delaware/22
100
A/laughing gull/Delaware/42
75
A/laughing gull/New Jersey/798/1986
49
A/Quail/Arkansas/29209-1/93
99
A/shorebird/Delaware/236/2003
89
A/shorebird/Delaware/10/2004
10

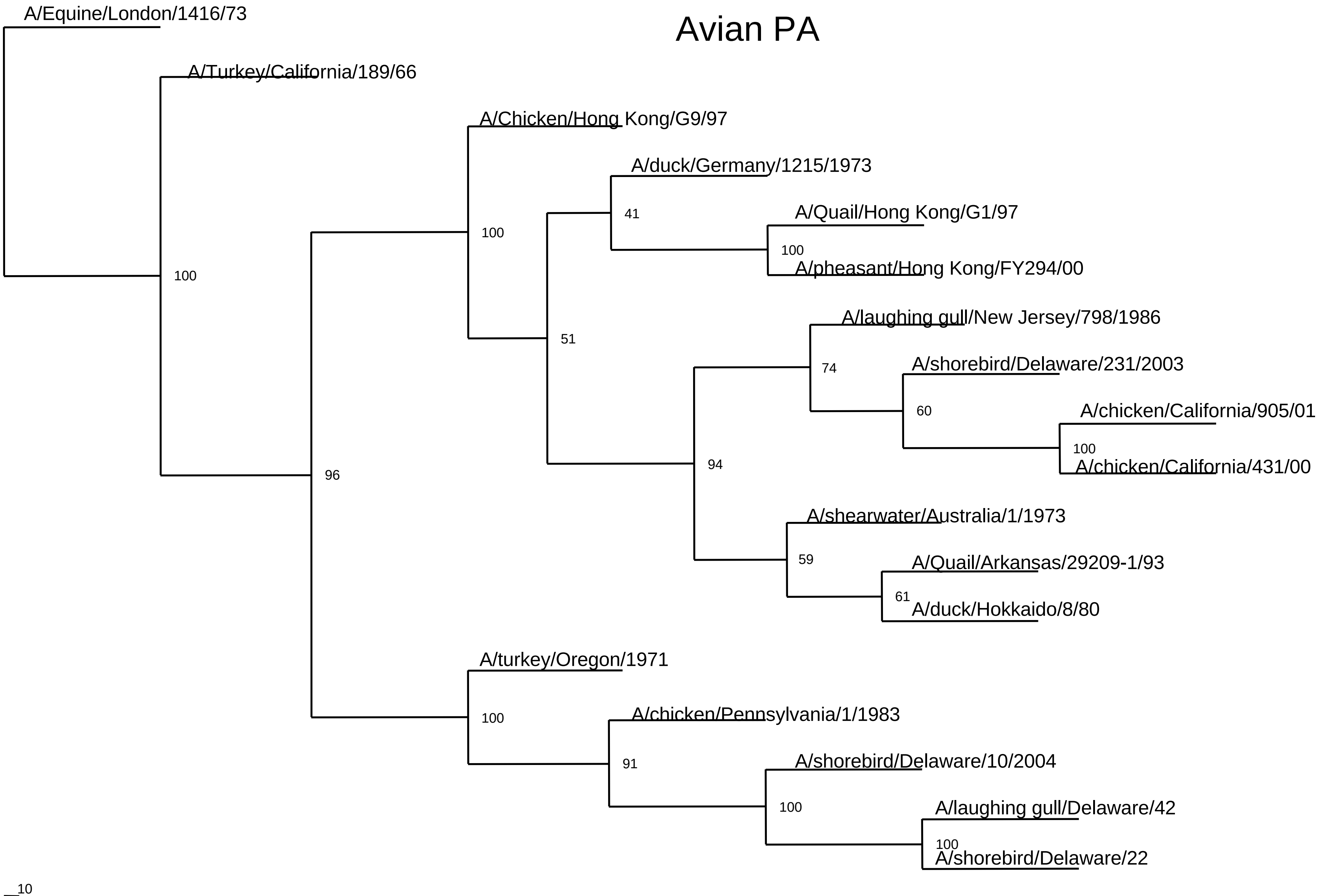

Avian PA
A/Equine/London/1416/73
A/Turkey/California/189/66
A/Chicken/Hong Kong/G9/97
A/duck/Germany/1215/1973
A/Quail/Hong Kong/G1/97
A/pheasant/Hong Kong/FY294/00
A/laughing gull/New Jersey/798/1986
A/shorebird/Delaware/231/2003
A/chicken/California/905/01
A/chicken/California/431/00
A/shearwater/Australia/1/1973
A/Quail/Arkansas/29209-1/93
A/duck/Hokkaido/8/80
A/turkey/Oregon/1971
A/chicken/Pennsylvania/1/1983
A/shorebird/Delaware/10/2004
A/laughing gull/Delaware/42
A/shorebird/Delaware/22
100
96
100
51
41
100
94
74
60
100
59
61
100
91
100
100
10

Avian PB1

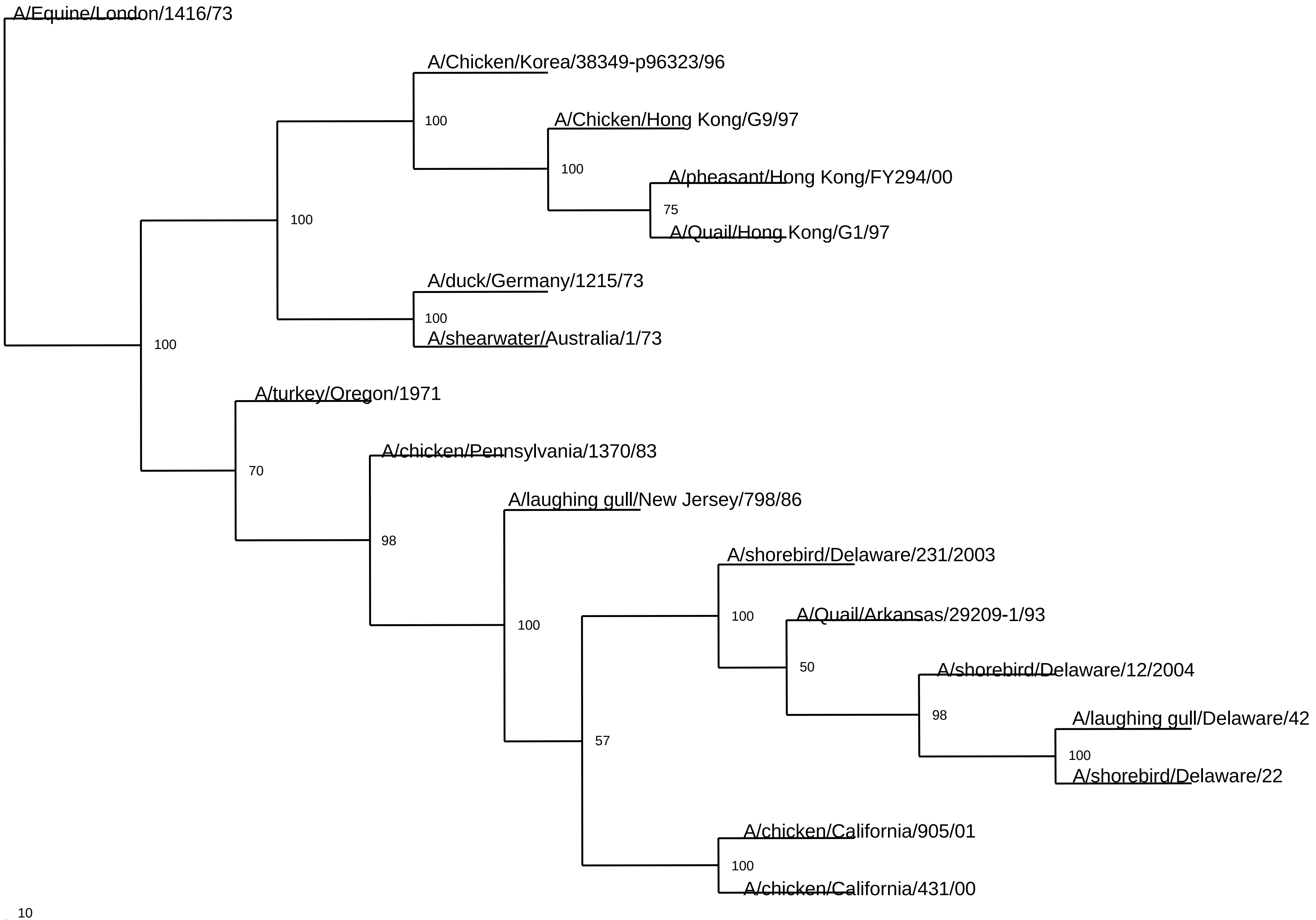
A/Equine/London/1416/73
A/Chicken/Korea/38349-p96323/96
A/Chicken/Hong Kong/G9/97
A/pheasant/Hong Kong/FY294/00
A/Quail/Hong Kong/G1/97
A/duck/Germany/1215/73
A/shearwater/Australia/1/73
A/turkey/Oregon/1971
A/chicken/Pennsylvania/1370/83
A/laughing gull/New Jersey/798/86
A/shorebird/Delaware/231/2003
A/Quail/Arkansas/29209-1/93
A/shorebird/Delaware/12/2004
A/laughing gull/Delaware/42
A/shorebird/Delaware/22
A/chicken/California/905/01
A/chicken/California/431/00
100
100
100
75
100
100
70
98
100
57
100
50
98
100
100
10

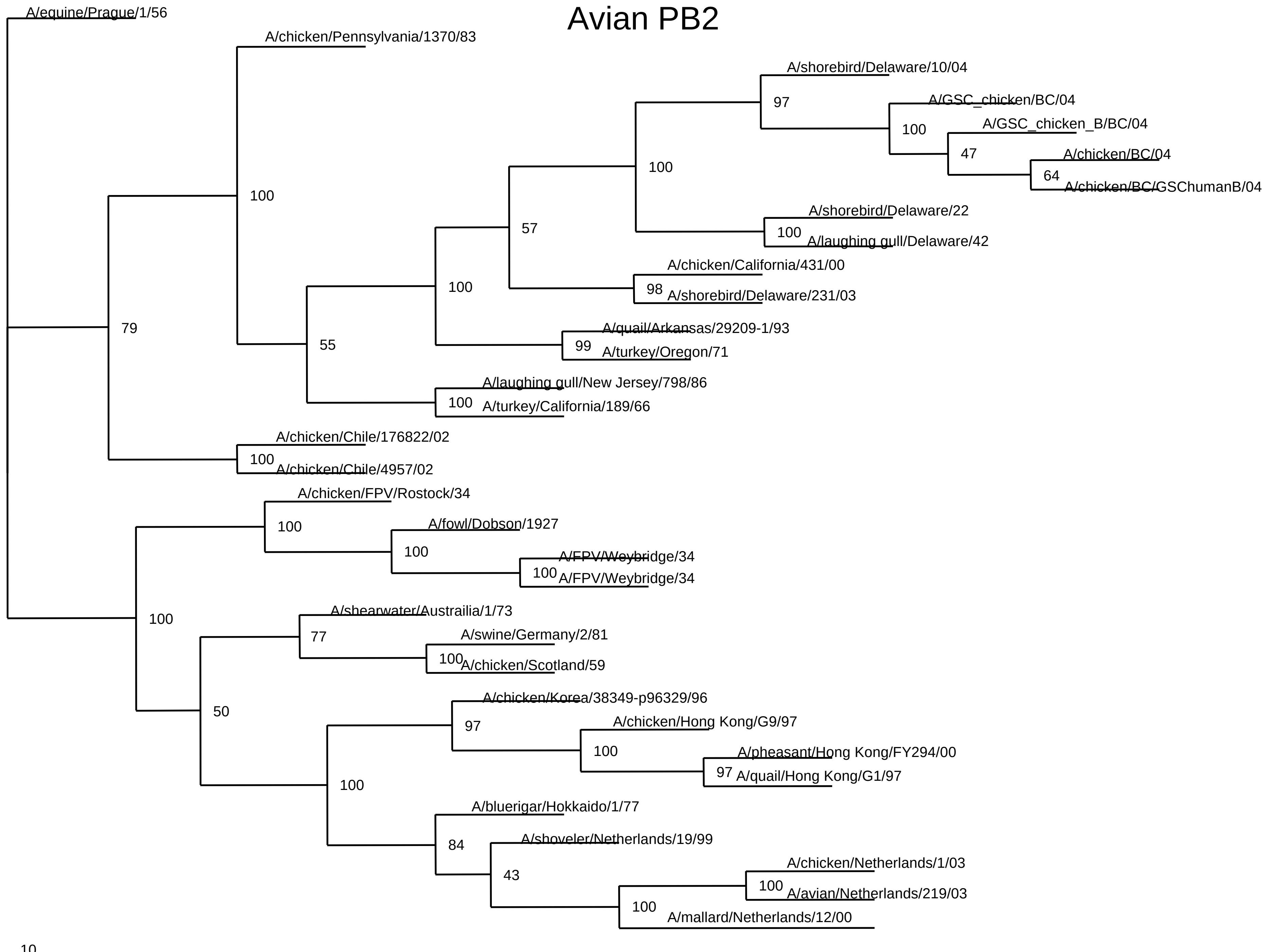

Avian PB2
A/equine/Prague/1/56
A/chicken/Pennsylvania/1370/83
A/shorebird/Delaware/10/04
A/GSC_chicken/BC/04
A/GSC_chicken_B/BC/04
A/chicken/BC/04
A/chicken/BC/GSChumanB/04
A/shorebird/Delaware/22
A/laughing gull/Delaware/42
A/chicken/California/431/00
A/shorebird/Delaware/231/03
A/quail/Arkansas/29209-1/93
A/turkey/Oregon/71
A/laughing gull/New Jersey/798/86
A/turkey/California/189/66
A/chicken/Chile/176822/02
A/chicken/Chile/4957/02
A/chicken/FPV/Rostock/34
A/fowl/Dobson/1927
A/FPV/Weybridge/34
A/FPV/Weybridge/34
A/shearwater/Austrailia/1/73
A/swine/Germany/2/81
A/chicken/Scotland/59
A/chicken/Korea/38349-p96329/96
A/chicken/Hong Kong/G9/97
A/pheasant/Hong Kong/FY294/00
A/quail/Hong Kong/G1/97
A/bluerigar/Hokkaido/1/77
A/shoveler/Netherlands/19/99
A/chicken/Netherlands/1/03
A/avian/Netherlands/219/03
A/mallard/Netherlands/12/00
97
100
47
64
100
100
57
98
100
99
55
100
100
79
100
100
100
100
100
77
100
50
97
100
97
100
84
43
100
100
10

Avian M

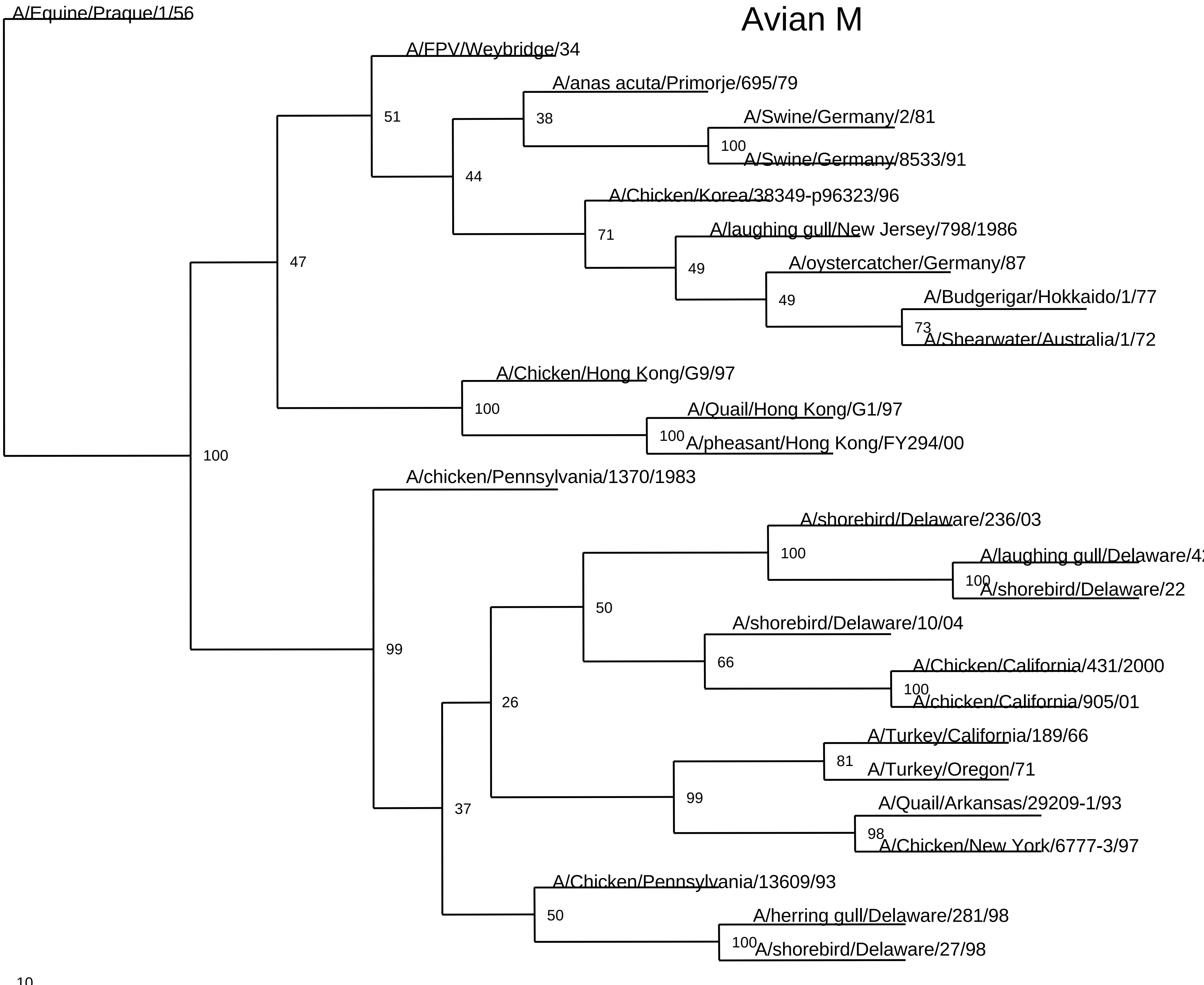
A/Equine/Praque/1/56
A/FPV/Weybridge/34
A/anas acuta/Primorje/695/79
A/Swine/Germany/2/81
A/Swine/Germany/8533/91
A/Chicken/Korea/38349-p96323/96
A/laughing gull/New Jersey/798/1986
A/oystercatcher/Germany/87
A/Budgerigar/Hokkaido/1/77
A/Shearwater/Australia/1/72
A/Chicken/Hong Kong/G9/97
A/Quail/Hong Kong/G1/97
A/pheasant/Hong Kong/FY294/00
A/chicken/Pennsylvania/1370/1983
A/shorebird/Delaware/236/03
A/laughing gull/Delaware/42
A/shorebird/Delaware/22
A/shorebird/Delaware/10/04
A/Chicken/California/431/2000
A/chicken/California/905/01
A/Turkey/California/189/66
A/Turkey/Oregon/71
A/Quail/Arkansas/29209-1/93
A/Chicken/New York/6777-3/97
A/Chicken/Pennsylvania/13609/93
A/herring gull/Delaware/281/98
A/shorebird/Delaware/27/98
51
38
100
44
71
49
49
73
47
100
100
100
99
100
100
50
66
100
26
81
99
98
37
50
100
10

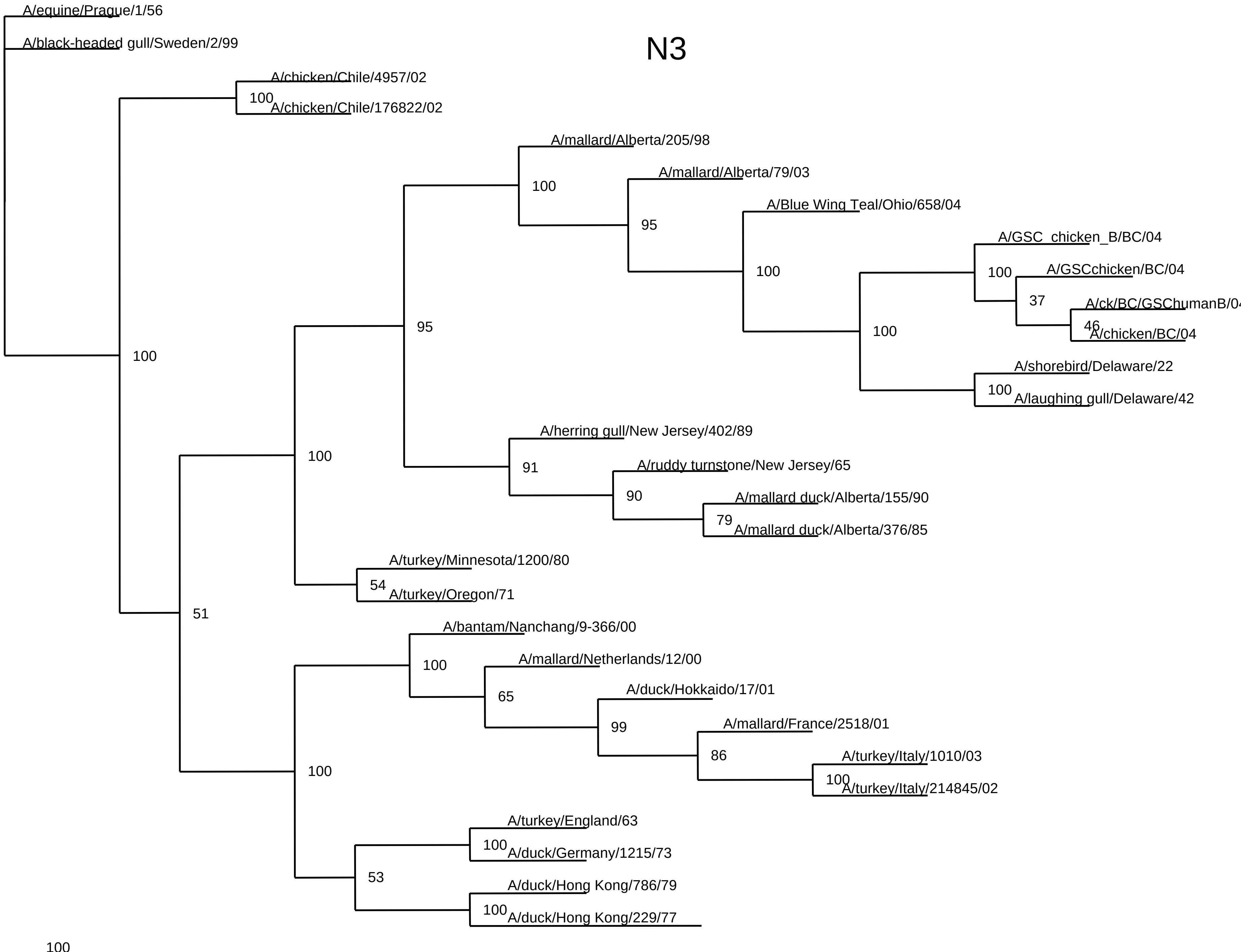

N3
A/equine/Prague/1/56
A/black-headed gull/Sweden/2/99
A/chicken/Chile/4957/02
A/chicken/Chile/176822/02
A/mallard/Alberta/205/98
A/mallard/Alberta/79/03
A/Blue Wing Teal/Ohio/658/04
A/GSC_chicken_B/BC/04
A/GSCchicken/BC/04
A/ck/BC/GSChumanB/04
A/chicken/BC/04
A/shorebird/Delaware/22
A/laughing gull/Delaware/42
A/herring gull/New Jersey/402/89
A/ruddy turnstone/New Jersey/65
A/mallard duck/Alberta/155/90
A/mallard duck/Alberta/376/85
A/turkey/Minnesota/1200/80
A/turkey/Oregon/71
A/bantam/Nanchang/9-366/00
A/mallard/Netherlands/12/00
A/duck/Hokkaido/17/01
A/mallard/France/2518/01
A/turkey/Italy/1010/03
A/turkey/Italy/214845/02
A/turkey/England/63
A/duck/Germany/1215/73
A/duck/Hong Kong/786/79
A/duck/Hong Kong/229/77
100
100
100
95
100
100
100
37
46
100
95
100
91
90
79
54
51
100
65
99
86
100
100
53
100
100
100

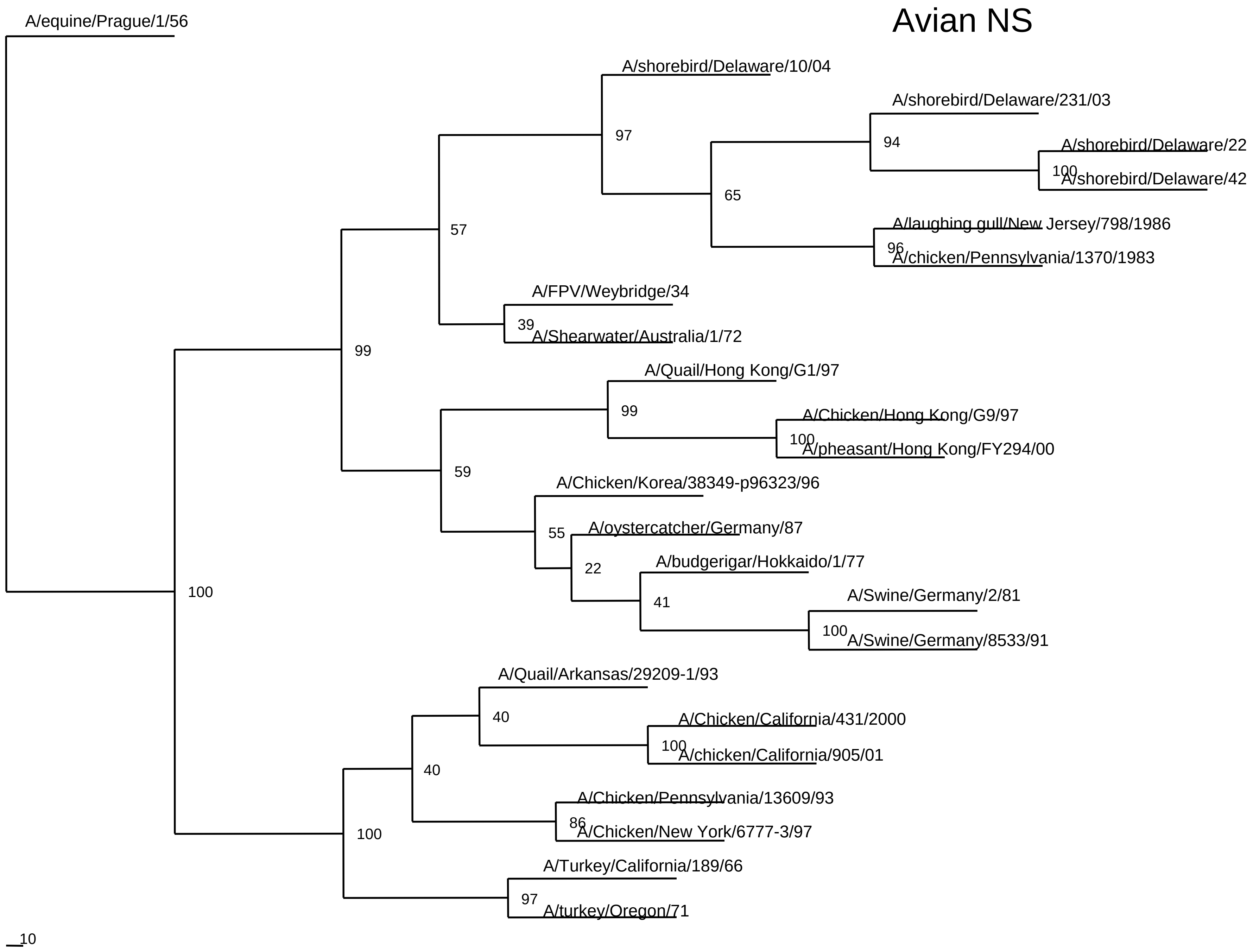
Avian NS
A/equine/Prague/1/56
A/shorebird/Delaware/10/04
A/shorebird/Delaware/231/03
A/shorebird/Delaware/22
A/shorebird/Delaware/42
A/laughing gull/New Jersey/798/1986
A/chicken/Pennsylvania/1370/1983
A/FPV/Weybridge/34
A/Shearwater/Australia/1/72
A/Quail/Hong Kong/G1/97
A/Chicken/Hong Kong/G9/97
A/pheasant/Hong Kong/FY294/00
A/Chicken/Korea/38349-p96323/96
A/oystercatcher/Germany/87
A/budgerigar/Hokkaido/1/77
A/Swine/Germany/2/81
A/Swine/Germany/8533/91
A/Quail/Arkansas/29209-1/93
A/Chicken/California/431/2000
A/chicken/California/905/01
A/Chicken/Pennsylvania/13609/93
A/Chicken/New York/6777-3/97
A/Turkey/California/189/66
A/turkey/Oregon/71
97
94
100
65
96
57
39
99
99
100
59
55
22
41
100
100
40
100
40
86
100
97
10
