# Supplementary material for: Influenza in Migratory Birds and Evidence of Limited Intercontinental Virus Exchange
Source: PLoS Pathog. 2007 Nov 9;3(11):e167. doi: 10.1371/journal.ppat.0030167 (PMC2065878; doi:10.1371/journal.ppat.0030167)
Supplement: Supplementary file 2 [file ppat.0030167.sg002.pdf]

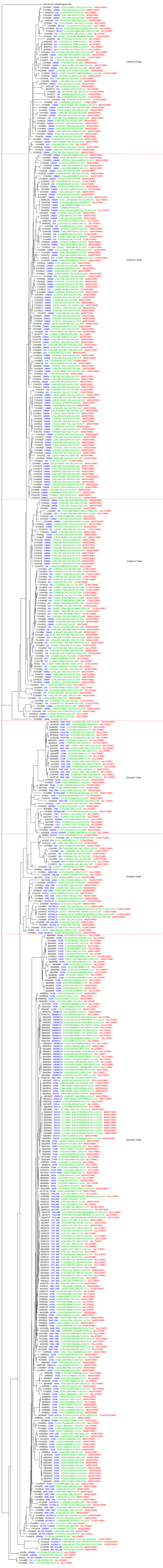

AAA43140 A/Eq/Prague/1/56
CY003967 CANADA A/BLUE-WINGED TEAL/ALB/16/1997 ANSERIFORMES
CY004514 CANADA A/MALLARD/ALB/201/1998 ANSERIFORMES
AY849783 USA A/CHICKEN/TX/298313/04 GALLIFORMES
CY003959 CANADA A/MALLARD/ALB/202/1996 ANSERIFORMES
CY004699 CANADA A/MALLARD/ALB/118/1995 ANSERIFORMES
CY014895 USA A/DUCK/NEW YORK/16873/1999 ANSERIFORMES
CY014887 USA A/CHICKEN/NEW YORK/14677-13/1998 GALLIFORMES
CY005844 MEXICO A/CHICKEN/CHIS/15224/1997 GALLIFORMES
CY005838 MEXICO A/CHICKEN/HIDALGO/28159-232/1994 GALLIFORMES
CY015103 MEXICO A/CHICKEN/QUERETARO/14588-19/1995 GALLIFORMES
CY014828 USA A/CHICKEN/NEW YORK/13828-3/1995 GALLIFORMES
CY015142 USA A/RUDDY TURNSTONE/DELAWARE/34/1993 CICONIIFORMES
AY849782 USA A/CHICKEN/TX/167280-4/02 GALLIFORMES
AF457672 USA A/CHICKEN/CALIFORNIA/905/01 GALLIFORMES
AF457689 USA A/CHICKEN/CALIFORNIA/465/00 GALLIFORMES
AF457714 USA A/CHICKEN/CALIFORNIA/1002A/00 GALLIFORMES
AF457697 USA A/CHICKEN/CALIFORNIA/431/00 GALLIFORMES
CY014836 USA A/GUINEA FOWL/NEW YORK/20221-11/1995 GALLIFORMES
CY014559 CANADA A/MALLARD DUCK/ALBERTA/323/1988 ANSERIFORMES
CY004867 USA A/RUDDY TURNSTONE/DE/512/1988 CICONIIFORMES
CY004874 USA A/KNOT/DE/541/1988 CICONIIFORMES
CY004918 CANADA A/MALLARD/ALB/49/1995 ANSERIFORMES
CY004910 CANADA A/BLUE-WINGED TEAL/ALB/293/1994 ANSERIFORMES
CY004538 CANADA A/MALLARD DUCK/ALB/322/1988 ANSERIFORMES
CY014555 CANADA A/MALLARD DUCK/ALBERTA/331/1985 ANSERIFORMES
CY005420 BRAZIL A/SEMI-PALMATED SANDPIPER/BRAZIL/43/1990 CICONIIFORMES
CY005091 CANADA A/PINTAIL/ALBERTA/129/1993 ANSERIFORMES
CY005085 USA A/RED KNOT/NJ/325/1989 CICONIIFORMES
CY003951 CANADA A/MALLARD/ALB/201/1996 ANSERIFORMES
CY005317 CANADA A/MALLARD/ALB/126/1991 ANSERIFORMES
AY233387 USA A/DUCK/NC/91347/01 ANSERIFORMES
DQ335778 USA A/TURKEY/OHIO/313053/04 GALLIFORMES
CY004427 USA A/LAUGHING GULL/DE/5/2003 CICONIIFORMES
CY005177 USA A/SHOREBIRD/DE/261/2003 CICONIIFORMES
CY005173 USA A/SHOREBIRD/DE/231/2003 CICONIIFORMES
CY005337 USA A/SHOREBIRD/DE/236/2003 CICONIIFORMES
CY005165 USA A/SHOREBIRD/DE/66/2003 CICONIIFORMES
CY004960 CANADA A/BLUE-WINGED TEAL/ALBERTA/293/2003 ANSERIFORMES
CY004896 CANADA A/BLUE-WINGED TEAL/ALB/103/1990 ANSERIFORMES
CY004891 CANADA A/BLUE-WINGED TEAL/ALB/67/1990 ANSERIFORMES
CY003943 CANADA A/MALLARD DUCK/ALB/353/1988 ANSERIFORMES
American Clade
American Clade
American Clade
Eurasian Clade
Eurasian Clade
Eurasian Clade
