# Supplementary material for: Influenza in Migratory Birds and Evidence of Limited Intercontinental Virus Exchange
Source: PLoS Pathog. 2007 Nov 9;3(11):e167. doi: 10.1371/journal.ppat.0030167 (PMC2065878; doi:10.1371/journal.ppat.0030167)
Supplement: Supplementary file 5 [file ppat.0030167.sg005.pdf]

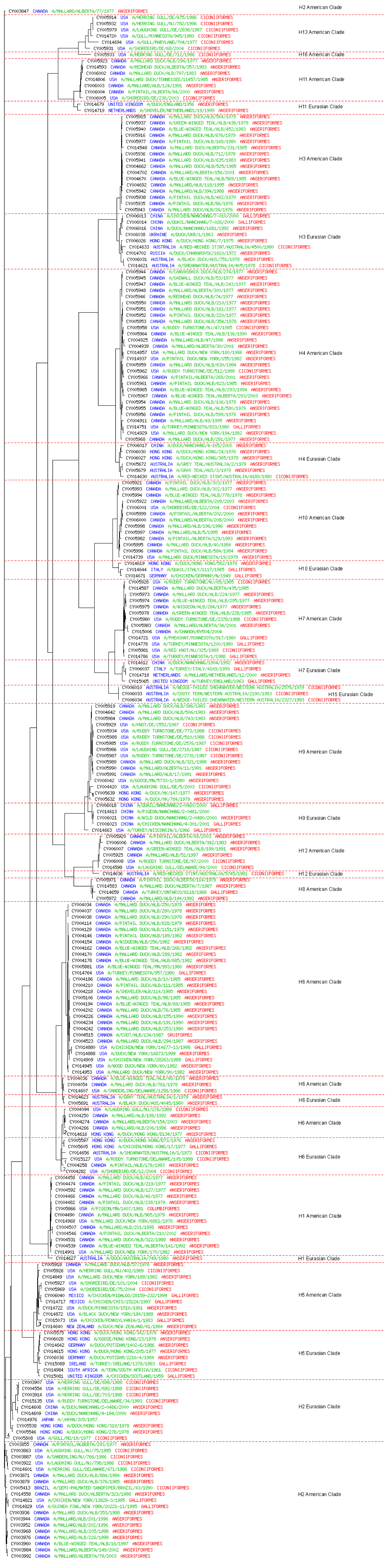

CY003847 CANADA A/MALLARD/ALBERTA/77/1977 ANSERIFORMES
H2 American Clade
CY005914 USA A/HERRING GULL/DE/475/1986 CICONIIFORMES
CY005932 USA A/HERRING GULL/NJ/782/1986 CICONIIFORMES
CY005979 USA A/LAUGHING GULL/DE/2838/1987 CICONIIFORMES
CY014720 USA A/GULL/MINNESOTA/945/1980 CICONIIFORMES
CY014694 USA A/GULL/MARYLAND/704/1977 CICONIIFORMES
CY005931 USA A/SHOREBIRD/DE/68/2004 CICONIIFORMES
H13 American Clade
CY005933 USA A/HERRING GULL/DE/712/1988 CICONIIFORMES
H16 American Clade
CY005923 CANADA A/MALLARD DUCK/ALB/294/1977 ANSERIFORMES
CY014593 CANADA A/REDHEAD DUCK/ALBERTA/357/1983 ANSERIFORMES
CY006002 CANADA A/MALLARD DUCK/ALB/797/1983 ANSERIFORMES
CY014806 USA A/MALLARD DUCK/TENNESSEE/11457/1985 ANSERIFORMES
CY006003 CANADA A/MALLARD/ALB/124/1991 ANSERIFORMES
CY006004 CANADA A/PINTAIL/ALBERTA/84/2000 ANSERIFORMES
CY006005 USA A/SHOREBIRD/DE/236/2003 CICONIIFORMES
H11 American Clade
CY014679 UNITED KINGDOM A/DUCK/ENGLAND/1956 ANSERIFORMES
CY014719 NETHERLANDS A/SHOVELER/NETHERLANDS/18/1999 ANSERIFORMES
H11 Eurasian Clade
CY005915 CANADA A/MALLARD DUCK/ALB/564/1979 ANSERIFORMES
CY005937 CANADA A/GREEN-WINGED TEAL/ALB/438/1979 ANSERIFORMES
CY005940 CANADA A/BLUE-WINGED TEAL/ALB/452/1983 ANSERIFORMES
CY005916 CANADA A/MALLARD DUCK/ALB/676/1979 ANSERIFORMES
CY005977 CANADA A/PINTAIL DUCK/ALB/169/1980 ANSERIFORMES
CY014548 CANADA A/MALLARD DUCK/ALBERTA/331/1985 ANSERIFORMES
CY005936 CANADA A/MALLARD DUCK/ALB/712/1978 ANSERIFORMES
CY005941 CANADA A/MALLARD DUCK/ALB/635/1983 ANSERIFORMES
CY004662 CANADA A/MALLARD DUCK/ALB/525/1985 ANSERIFORMES
CY004702 CANADA A/MALLARD/ALBERTA/156/2001 ANSERIFORMES
CY004670 CANADA A/BLUE-WINGED TEAL/ALB/569/1985 ANSERIFORMES
CY004692 CANADA A/MALLARD/ALB/118/1995 ANSERIFORMES
CY005942 CANADA A/MALLARD/ALB/394/1988 ANSERIFORMES
CY005938 CANADA A/PINTAIL DUCK/ALB/462/1979 ANSERIFORMES
CY005935 CANADA A/PINTAIL DUCK/ALB/86/1976 ANSERIFORMES
CY005943 CANADA A/MALLARD DUCK/ALB/26/1976 ANSERIFORMES
H3 American Clade
CY006013 CHINA A/CHICKEN/NANCHANG/7-010/2000 GALLIFORMES
CY006014 CHINA A/QUAIL/NANCHANG/7-026/2000 GALLIFORMES
CY006016 CHINA A/DUCK/NANCHANG/1681/1992 ANSERIFORMES
CY006038 UKRAINE A/DUCK/UKR/1/1963 ANSERIFORMES
CY006026 HONG KONG A/DUCK/HONG KONG/7/1975 ANSERIFORMES
CY014633 AUSTRALIA A/RED-NECKED STINT/AUSTRALIA/4500/1980 CICONIIFORMES
CY014702 RUSSIA A/DUCK/CHABAROVSK/1610/1972 ANSERIFORMES
CY006031 AUSTRALIA A/BLACK DUCK/AUS/751/1978 ANSERIFORMES
CY014621 AUSTRALIA A/SHEARWATER/AUSTRALIA/405/1978 CICONIIFORMES
H3 Eurasian Clade
CY005944 CANADA A/CANVASBACK DUCK/ALB/274/1977 ANSERIFORMES
CY005945 CANADA A/GADWALL DUCK/ALB/53/1977 ANSERIFORMES
CY005947 CANADA A/BLUE-WINGED TEAL/ALB/243/1977 ANSERIFORMES
CY005948 CANADA A/MALLARD/ALBERTA/300/1977 ANSERIFORMES
CY005946 CANADA A/REDHEAD DUCK/ALB/74/1977 ANSERIFORMES
CY005950 CANADA A/MALLARD DUCK/ALB/210/1977 ANSERIFORMES
CY005951 CANADA A/MALLARD DUCK/ALB/161/1977 ANSERIFORMES
CY005952 CANADA A/PINTAIL DUCK/ALB/220/1977 ANSERIFORMES
CY005953 CANADA A/MALLARD DUCK/ALB/354/1978 ANSERIFORMES
CY005958 USA A/RUDDY TURNSTONE/NJ/47/1985 CICONIIFORMES
CY005964 CANADA A/BLUE-WINGED TEAL/ALB/136/1990 ANSERIFORMES
CY004925 CANADA A/MALLARD/ALB/47/1998 ANSERIFORMES
CY004939 CANADA A/MALLARD/ALBERTA/30/2001 ANSERIFORMES
CY014857 USA A/MALLARD DUCK/NEW YORK/180/1986 ANSERIFORMES
CY014937 USA A/PINTAIL DUCK/NEW YORK/155/1982 ANSERIFORMES
CY005959 CANADA A/MALLARD DUCK/ALB/630/1984 ANSERIFORMES
CY005962 USA A/RUDDY TURNSTONE/DE/512/1986 CICONIIFORMES
CY005966 CANADA A/MALLARD/ALBERTA/269/2001 ANSERIFORMES
CY005961 CANADA A/PINTAIL DUCK/ALB/623/1985 ANSERIFORMES
CY005965 CANADA A/BLUE-WINGED TEAL/ALB/293/1993 ANSERIFORMES
CY005967 CANADA A/BLUE-WINGED TEAL/ALBERTA/293/2003 ANSERIFORMES
CY005954 CANADA A/MALLARD DUCK/ALB/106/1979 ANSERIFORMES
CY005955 CANADA A/BLUE-WINGED TEAL/ALB/580/1979 ANSERIFORMES
CY005956 CANADA A/PINTAIL DUCK/ALB/599/1979 ANSERIFORMES
CY004911 CANADA A/MALLARD/ALB/49/1995 ANSERIFORMES
CY014751 USA A/TURKEY/MINNESOTA/833/1980 GALLIFORMES
CY014929 USA A/MALLARD DUCK/NEW YORK/194/1982 ANSERIFORMES
CY005968 CANADA A/MALLARD DUCK/ALB/291/1977 ANSERIFORMES
H4 American Clade
CY006017 CHINA A/DUCK/NANCHANG/4-165/2000 ANSERIFORMES
CY006030 HONG KONG A/DUCK/HONG KONG/24/1976 ANSERIFORMES
CY006027 HONG KONG A/DUCK/HONG KONG/365/1978 ANSERIFORMES
CY005672 AUSTRALIA A/GREY TEAL/AUSTRALIA/2/1979 ANSERIFORMES
CY005679 AUSTRALIA A/GRAY TEAL/AUS/3/1979 ANSERIFORMES
CY014630 AUSTRALIA A/RED-NECKED STINT/AUSTRALIA/4189/1980 CICONIIFORMES
H4 Eurasian Clade
CY005921 CANADA A/PINTAIL DUCK/ALB/303/1977 ANSERIFORMES
CY005993 CANADA A/MALLARD DUCK/ALB/302/1977 ANSERIFORMES
CY005994 CANADA A/BLUE-WINGED TEAL/ALB/778/1978 ANSERIFORMES
CY005922 CANADA A/MALLARD/ALBERTA/209/2003 ANSERIFORMES
CY006001 USA A/SHOREBIRD/DE/122/2004 CICONIIFORMES
CY005999 CANADA A/PINTAIL/ALBERTA/202/2000 ANSERIFORMES
CY006000 CANADA A/MALLARD/ALBERTA/208/2000 ANSERIFORMES
CY005998 CANADA A/MALLARD/ALB/196/1996 ANSERIFORMES
CY005997 CANADA A/MALLARD/ALB/5/1995 ANSERIFORMES
CY005982 CANADA A/PINTAIL/ALBERTA/129/1993 ANSERIFORMES
CY005995 CANADA A/MALLARD DUCK/ALB/40/1984 ANSERIFORMES
CY005996 CANADA A/PINTAIL DUCK/ALB/584/1984 ANSERIFORMES
CY014739 USA A/MALLARD DUCK/MINNESOTA/19/1979 ANSERIFORMES
H10 American Clade
CY014619 HONG KONG A/DUCK/HONG KONG/562/1979 ANSERIFORMES
CY014644 ITALY A/QUAIL/ITALY/1117/1965 GALLIFORMES
CY014671 GERMANY A/CHICKEN/GERMANY/N/1949 GALLIFORMES
H10 Eurasian Clade
CY005928 USA A/RUDDY TURNSTONE/NJ/65/1985 CICONIIFORMES
CY014587 CANADA A/MALLARD DUCK/ALBERTA/435/1985
CY005973 CANADA A/MALLARD DUCK/ALB/224/1977 ANSERIFORMES
CY005974 CANADA A/BLUE-WINGED TEAL/ALB/295/1977 ANSERIFORMES
CY005975 CANADA A/WIDGEON/ALB/284/1977 ANSERIFORMES
CY005978 CANADA A/GREEN-WINGED TEAL/ALB/228/1985 ANSERIFORMES
CY005980 USA A/RUDDY TURNSTONE/DE/2378/1988 CICONIIFORMES
CY005983 CANADA A/MALLARD/ALBERTA/34/2001 ANSERIFORMES
CY015006 CANADA A/CANADA/RV504/2004
CY014721 USA A/PHEASANT/MINNESOTA/917/1980 GALLIFORMES
CY014778 USA A/TURKEY/MINNESOTA/1200/1980 GALLIFORMES
CY005981 USA A/RED KNOT/NJ/325/1989 CICONIIFORMES
CY014786 USA A/TURKEY/MINNESOTA/1/1988 GALLIFORMES
H7 American Clade
CY014612 CHINA A/DUCK/NANCHANG/1904/1992 ANSERIFORMES
CY006037 ITALY A/TURKEY/ITALY/4169/1999 GALLIFORMES
CY014718 NETHERLANDS A/MALLARD/NETHERLANDS/12/2000 ANSERIFORMES
CY015065 UNITED KINGDOM A/TURKEY/ENGLAND/1963 GALLIFORMES
H7 Eurasian Clade
CY006010 AUSTRALIA A/WEDGE-TAILED SHEARWATER/WESTERN AUSTRALIA/2576/1979 CICONIIFORMES
CY006033 AUSTRALIA A/SOOTY TERN/WESTERN AUSTRALIA/2190/1983 CICONIIFORMES
CY006034 AUSTRALIA A/WEDGE-TAILED SHEARWATER/WESTERN AUSTRALIA/2327/1983 CICONIIFORMES
H15 Eurasian Clade
CY005919 CANADA A/MALLARD DUCK/ALB/396/1983 ANSERIFORMES
CY004642 CANADA A/MALLARD DUCK/ALB/506/1983 ANSERIFORMES
CY005984 CANADA A/MALLARD DUCK/ALB/743/1983 ANSERIFORMES
CY005929 USA A/KNOT/DE/2552/1987 CICONIIFORMES
CY005934 USA A/RUDDY TURNSTONE/DE/773/1988 CICONIIFORMES
CY005988 USA A/RUDDY TURNSTONE/DE/510/1988 CICONIIFORMES
CY005985 USA A/RUDDY TURNSTONE/DE/2576/1987 CICONIIFORMES
CY005986 USA A/LAUGHING GULL/DE/2718/1987 CICONIIFORMES
CY005987 USA A/RUDDY TURNSTONE/DE/2731/1987 CICONIIFORMES
CY005989 CANADA A/MALLARD DUCK/ALB/321/1988 ANSERIFORMES
CY005990 CANADA A/MALLARD/ALBERTA/11/1991 ANSERIFORMES
CY005991 CANADA A/MALLARD/ALB/17/1991 ANSERIFORMES
CY006042 USA A/GOOSE/MN/5733-1/1980 ANSERIFORMES
CY004420 USA A/LAUGHING GULL/DE/5/2003 CICONIIFORMES
1
CY005639 HONG KONG A/DUCK/HK/147/1977 ANSERIFORMES
CY005632 HONG KONG A/DUCK/HK/784/1979 ANSERIFORMES
H9 American Clade
CY006018 CHINA A/QUAIL/NANCHANG/2-0460/2000 GALLIFORMES
CY014613 CHINA A/PIGEON/NANCHANG/2-0461/2000
CY006021 CHINA A/WILD DUCK/NANCHANG/2-0480/2000 ANSERIFORMES
CY006023 CHINA A/CHICKEN/NANCHANG/4-301/2001 GALLIFORMES
CY014663 USA A/TURKEY/WISCONSIN/1/1966 GALLIFORMES
H9 Eurasian Clade
CY005920 CANADA A/PINTAIL/ALBERTA/49/2003 ANSERIFORMES
CY006006 CANADA A/MALLARD DUCK/ALBERTA/342/1983 ANSERIFORMES
CY006007 CANADA A/GREEN-WINGED TEAL/ALB/199/1991 ANSERIFORMES
CY005925 CANADA A/MALLARD/ALB/52/1997 ANSERIFORMES
CY006008 USA A/RUDDY TURNSTONE/DE/97/2000 CICONIIFORMES
CY014598 USA A/LAUGHING GULL/DELAWARE/94/2000 CICONIIFORMES
H12 American Clade
CY014636 AUSTRALIA A/RED-NECKED STINT/AUSTRALIA/5745/1981 CICONIIFORMES
H12 Eurasian Clade
CY005971 CANADA A/PINTAIL DUCK/ALBERTA/114/1979 ANSERIFORMES
CY014583 CANADA A/MALLARD DUCK/ALBERTA/7/1987 ANSERIFORMES
CY014659 CANADA A/TURKEY/ONTARIO/6118/1968 GALLIFORMES
CY005972 CANADA A/MALLARD/ALB/194/1992 ANSERIFORMES
H8 American Clade
CY004034 CANADA A/MALLARD DUCK/ALB/250/1978 ANSERIFORMES
CY004037 CANADA A/MALLARD DUCK/ALB/280/1978 ANSERIFORMES
CY004038 CANADA A/MALLARD DUCK/ALB/290/1978 ANSERIFORMES
CY004114 CANADA A/PINTAIL DUCK/ALB/628/1979 ANSERIFORMES
CY004129 CANADA A/MALLARD DUCK/ALB/1151/1979 ANSERIFORMES
CY004146 CANADA A/PINTAIL DUCK/ALB/189/1982 ANSERIFORMES
CY004154 CANADA A/WIDGEON/ALB/256/1982 ANSERIFORMES
CY004162 CANADA A/BLUE-WINGED TEAL/ALB/266/1982 ANSERIFORMES
CY004170 CANADA A/MALLARD DUCK/ALB/289/1982 ANSERIFORMES
CY004178 CANADA A/BLUE-WINGED TEAL/ALB/685/1982 ANSERIFORMES
CY005881 USA A/BLUE-WINGED TEAL/MN/993/1980 ANSERIFORMES
CY014764 USA A/TURKEY/MINNESOTA/957/1980 GALLIFORMES
CY004186 CANADA A/MALLARD DUCK/ALB/10/1985 ANSERIFORMES
CY004210 CANADA A/PINTAIL DUCK/ALB/111/1985 ANSERIFORMES
CY004218 CANADA A/SHOVELER/ALB/114/1985 ANSERIFORMES
CY005106 CANADA A/MALLARD DUCK/ALB/98/1985 ANSERIFORMES
CY004194 CANADA A/BLUE-WINGED TEAL/ALB/69/1985 ANSERIFORMES
CY004202 CANADA A/MALLARD DUCK/ALB/76/1985 ANSERIFORMES
CY004226 CANADA A/MALLARD DUCK/ALB/155/1990 ANSERIFORMES
CY004234 CANADA A/MALLARD DUCK/ALB/191/1990 ANSERIFORMES
CY004242 CANADA A/MALLARD DUCK/ALB/253/1990 ANSERIFORMES
CY004515 CANADA A/COOT/ALB/134/1987 GRUIFORMES
CY004523 CANADA A/MALLARD DUCK/ALB/294/1987 ANSERIFORMES
CY014880 USA A/CHICKEN/NEW YORK/14677-13/1998 GALLIFORMES
CY014888 USA A/DUCK/NEW YORK/16873/1999 ANSERIFORMES
CY014909 USA A/CHICKEN/NEW YORK/28263/1989 GALLIFORMES
CY014945 USA A/WOOD DUCK/NEW YORK/60/1982 ANSERIFORMES
CY014953 USA A/MALLARD DUCK/NEW YORK/90/1982 ANSERIFORMES
H6 American Clade
CY004036 CANADA A/BLUE-WINGED TEAL/ALB/48/1978 ANSERIFORMES
CY004054 CANADA A/MALLARD DUCK/ALB/761/1978 ANSERIFORMES
CY014607 USA A/SANDERLING/DELAWARE/1258/1986 CICONIIFORMES
H6 American Clade
CY014623 AUSTRALIA A/GRAY TEAL/AUSTRALIA/1/1979 ANSERIFORMES
CY005691 AUSTRALIA A/BLACK DUCK/AUS/4045/1980 ANSERIFORMES
H6 Eurasian Clade
CY004094 USA A/LAUGHING GULL/NJ/276/1989 CICONIIFORMES
CY004250 CANADA A/MALLARD/ALB/199/1992 ANSERIFORMES
CY004274 CANADA A/MALLARD/ALBERTA/154/2003 ANSERIFORMES
CY004266 CANADA A/MALLARD/ALB/206/1996 ANSERIFORMES
2
CY014616 HONG KONG A/DUCK/HONG KONG/D134/1977 ANSERIFORMES
H6 American Clade
CY005597 HONG KONG A/DUCK/HONG KONG/D73/1976 ANSERIFORMES
CY005605 HONG KONG A/CHICKEN/HONG KONG/17/1977 GALLIFORMES
CY014656 AUSTRALIA A/SHEARWATER/AUSTRALIA/1/1973 CICONIIFORMES
CY015127 USA A/RUDDY TURNSTONE/DELAWARE/105/1998 CICONIIFORMES
1
CY004258 CANADA A/PINTAIL/ALB/179/1993 ANSERIFORMES
2
CY004282 USA A/SHOREBIRD/DE/12/2004 CICONIIFORMES
H6 Eurasian Clade
CY004458 CANADA A/MALLARD DUCK/ALB/42/1977 ANSERIFORMES
CY004474 CANADA A/PINTAIL DUCK/ALB/219/1977 ANSERIFORMES
CY004592 CANADA A/MALLARD DUCK/ALB/127/1977 ANSERIFORMES
CY004466 CANADA A/MALLARD DUCK/ALB/46/1977 ANSERIFORMES
CY004482 CANADA A/PINTAIL DUCK/ALB/238/1979 ANSERIFORMES
CY005866 USA A/PIGEON/MN/1407/1981 COLUMBIFORMES
CY004490 CANADA A/MALLARD DUCK/ALB/965/1979 ANSERIFORMES
CY014968 USA A/MALLARD DUCK/NEW YORK/6861/1978 ANSERIFORMES
CY004507 CANADA A/MALLARD/ALB/201/1998 ANSERIFORMES
CY004546 CANADA A/PINTAIL DUCK/ALBERTA/210/2002 ANSERIFORMES
CY004531 CANADA A/MALLARD DUCK/ALB/322/1988 ANSERIFORMES
CY004539 CANADA A/BLUE-WINGED TEAL/ALBERTA/141/1992 ANSERIFORMES
CY014901 USA A/MALLARD DUCK/NEW YORK/170/1982 ANSERIFORMES
H1 American Clade
CY014627 AUSTRALIA A/DUCK/AUSTRALIA/749/1980 ANSERIFORMES
H1 Eurasian Clade
CY005918 CANADA A/MALLARD DUCK/ALB/57/1976 ANSERIFORMES
CY005926 USA A/HERRING GULL/NJ/402/1989 CICONIIFORMES
CY014849 USA A/MALLARD DUCK/NEW YORK/189/1982 ANSERIFORMES
CY005927 USA A/SHOREBIRD/DE/101/2004 CICONIIFORMES
CY005969 USA A/SHOREBIRD/DE/75/2004 CICONIIFORMES
CY006040 MEXICO A/CHICKEN/HIDALGO/28159-232/1994 GALLIFORMES
CY014717 MEXICO A/CHICKEN/CHIS/15224/1997 GALLIFORMES
CY014722 USA A/DUCK/MINNESOTA/1516/1981 ANSERIFORMES
CY014872 USA A/BLACK DUCK/NEW YORK/184/1988 ANSERIFORMES
CY015073 USA A/CHICKEN/PENNSYLVANIA/1/1983 GALLIFORMES
3
CY014640 NEW ZEALAND A/DUCK/NEW ZEALAND/41/1984 ANSERIFORMES
H5 American Clade
CY005575 HONG KONG A/DUCK/HONG KONG/342/1978 ANSERIFORMES
CY006028 HONG KONG A/GOOSE/HONG KONG/23/1978 ANSERIFORMES
CY014642 GERMANY A/DUCK/POTSDAM/1402-6/1986 ANSERIFORMES
CY014615 HONG KONG A/DUCK/HONG KONG/205/1977 ANSERIFORMES
CY006036 GERMANY A/DUCK/POTSDAM/2216-4/1984 ANSERIFORMES
CY015089 IRELAND A/TURKEY/IRELAND/1378/1983 GALLIFORMES
CY014984 SOUTH AFRICA A/TERN/SOUTH AFRICA/1961 CICONIIFORMES
CY015081 UNITED KINGDOM A/CHICKEN/SCOTLAND/1959 GALLIFORMES
H5 Eurasian Clade
CY003907 USA A/HERRING GULL/DE/698/1988 CICONIIFORMES
3
CY004554 USA A/HERRING GULL/DE/692/1988 CICONIIFORMES
CY003914 USA A/HERRING GULL/DE/703/1988 CICONIIFORMES
CY015135 USA A/RUDDY TURNSTONE/DELAWARE/34/1993 CICONIIFORMES
CY014608 CHINA A/DUCK/NANCHANG/2-0486/2000 ANSERIFORMES
CY014609 CHINA A/DUCK/NANCHANG/4-184/2000 ANSERIFORMES
CY014976 JAPAN A/JAPAN/305/1957
CY005538 HONG KONG A/DUCK/HONG KONG/319/1978 ANSERIFORMES
CY005546 HONG KONG A/DUCK/HONG KONG/278/1978 ANSERIFORMES
4
CY005808 USA A/GULL/MD/19/1977 CICONIIFORMES
H2 Eurasian Clade
CY003855 CANADA A/PINTAIL/ALBERTA/293/1977 ANSERIFORMES
CY003863 USA A/LAUGHING GULL/NJ/75/1985 CICONIIFORMES
CY003887 USA A/SANDERLING/NJ/766/1986 CICONIIFORMES
CY003922 USA A/LAUGHING GULL/NJ/798/1986 CICONIIFORMES
CY014601 USA A/HERRING GULL/DELAWARE/471/1986 CICONIIFORMES
CY003871 CANADA A/MALLARD DUCK/ALB/884/1984 ANSERIFORMES
CY003879 CANADA A/MALLARD DUCK/ALB/376/1985 ANSERIFORMES
CY005413 BRAZIL A/SEMI-PALMATED SANDPIPER/BRAZIL/43/1990 CICONIIFORMES
CY014558 CANADA A/MALLARD DUCK/ALBERTA/323/1988 ANSERIFORMES
CY014821 USA A/CHICKEN/NEW YORK/13828-3/1995 GALLIFORMES
CY014829 USA A/GUINEA FOWL/NEW YORK/20221-11/1995 GALLIFORMES
CY003936 CANADA A/MALLARD DUCK/ALB/353/1988 ANSERIFORMES
CY003944 CANADA A/MALLARD/ALB/201/1996 ANSERIFORMES
CY003952 CANADA A/MALLARD/ALB/202/1996 ANSERIFORMES
CY003968 CANADA A/MALLARD/ALB/205/1998 ANSERIFORMES
CY003976 CANADA A/MALLARD/ALB/226/1998 ANSERIFORMES
CY003960 CANADA A/BLUE-WINGED TEAL/ALB/16/1997 ANSERIFORMES
CY003984 CANADA A/MALLARD/ALBERTA/149/2002 ANSERIFORMES
CY003992 CANADA A/MALLARD/ALBERTA/79/2003 ANSERIFORMES
H2 American Clade
