# Supplementary material for: Influenza in Migratory Birds and Evidence of Limited Intercontinental Virus Exchange
Source: PLoS Pathog. 2007 Nov 9;3(11):e167. doi: 10.1371/journal.ppat.0030167 (PMC2065878; doi:10.1371/journal.ppat.0030167)
Supplement: Supplementary file 6 [file ppat.0030167.sg006.pdf]

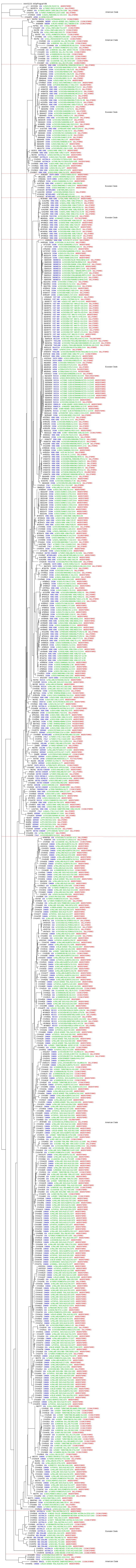
AAA52233 A/Eq/Prague/1/56
American Clade
American Clade
Eurasian Clade
Eurasian Clade
Eurasian Clade
Eurasian Clade
American Clade
Eurasian Clade
