# Supplementary material for: Influenza in Migratory Birds and Evidence of Limited Intercontinental Virus Exchange
Source: PLoS Pathog. 2007 Nov 9;3(11):e167. doi: 10.1371/journal.ppat.0030167 (PMC2065878; doi:10.1371/journal.ppat.0030167)
Supplement: Supplementary file 11 [file ppat.0030167.sg011.pdf]

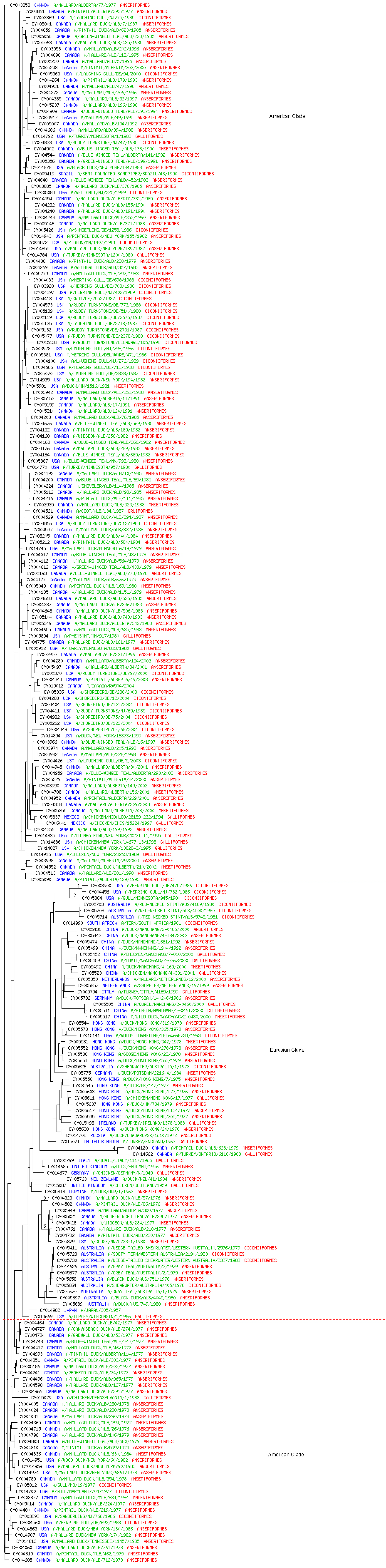

American Clade
Eurasian Clade
American Clade
