# Supplementary material for: Influenza in Migratory Birds and Evidence of Limited Intercontinental Virus Exchange
Source: PLoS Pathog. 2007 Nov 9;3(11):e167. doi: 10.1371/journal.ppat.0030167 (PMC2065878; doi:10.1371/journal.ppat.0030167)
Supplement: Supplementary file 13 [file ppat.0030167.sg013.pdf]

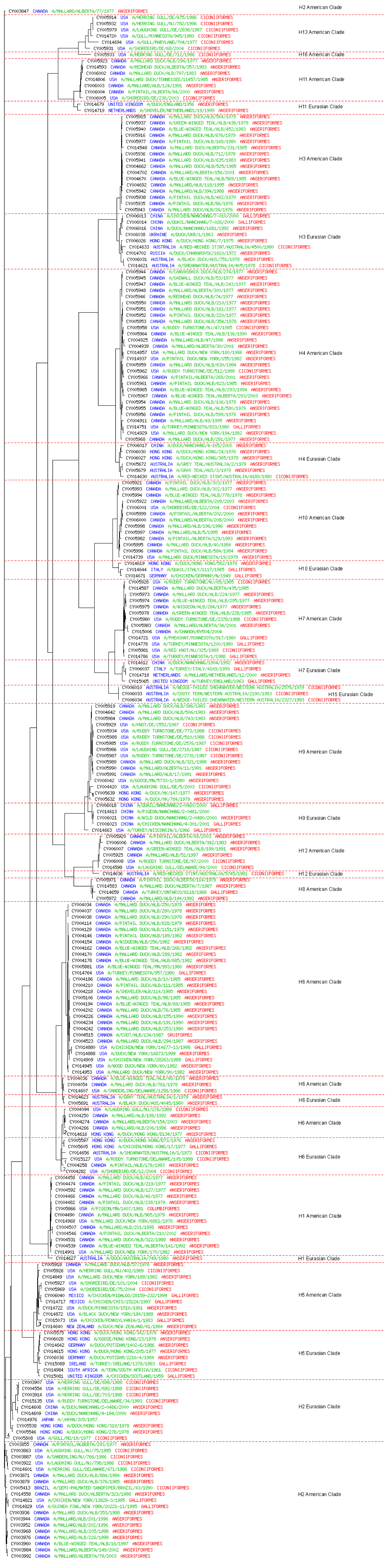

H2 American Clade
H13 American Clade
H16 American Clade
H11 American Clade
H11 Eurasian Clade
H3 American Clade
H3 Eurasian Clade
H4 American Clade
H4 Eurasian Clade
H10 American Clade
H10 Eurasian Clade
H7 American Clade
H7 Eurasian Clade
H15 Eurasian Clade
H9 American Clade
H9 Eurasian Clade
H12 American Clade
H12 Eurasian Clade
H8 American Clade
H6 American Clade
H6 American Clade
H6 Eurasian Clade
H6 American Clade
H6 Eurasian Clade
H1 American Clade
H1 Eurasian Clade
H5 American Clade
H5 Eurasian Clade
H2 Eurasian Clade
H2 American Clade
