# Supplementary material for: Influenza in Migratory Birds and Evidence of Limited Intercontinental Virus Exchange
Source: PLoS Pathog. 2007 Nov 9;3(11):e167. doi: 10.1371/journal.ppat.0030167 (PMC2065878; doi:10.1371/journal.ppat.0030167)
Supplement: Supplementary file 15 [file ppat.0030167.sg015.pdf]

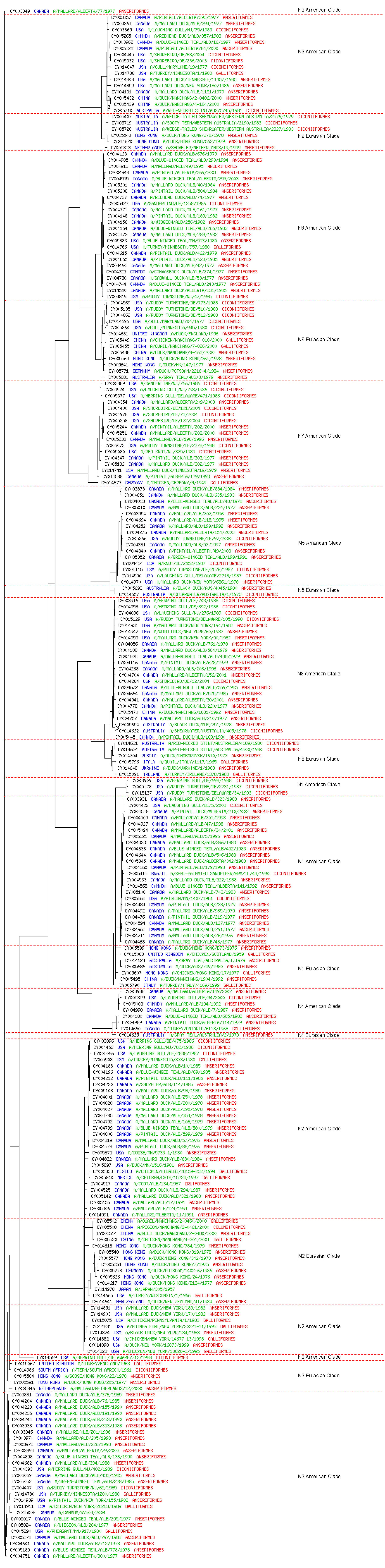
N3 American Clade
N9 American Clade
N9 Eurasian Clade
N6 American Clade
N6 Eurasian Clade
N7 American Clade
N5 American Clade
N5 Eurasian Clade
N8 American Clade
N8 Eurasian Clade
N1 American Clade
N1 American Clade
N1 Eurasian Clade
N4 American Clade
N4 Eurasian Clade
N2 American Clade
N2 Eurasian Clade
N2 American Clade
N3 American Clade
N3 Eurasian Clade
N3 American Clade
