# Supplementary material for: Influenza in Migratory Birds and Evidence of Limited Intercontinental Virus Exchange
Source: PLoS Pathog. 2007 Nov 9;3(11):e167. doi: 10.1371/journal.ppat.0030167 (PMC2065878; doi:10.1371/journal.ppat.0030167)
Supplement: Supplementary file 16 [file ppat.0030167.sg016.pdf]

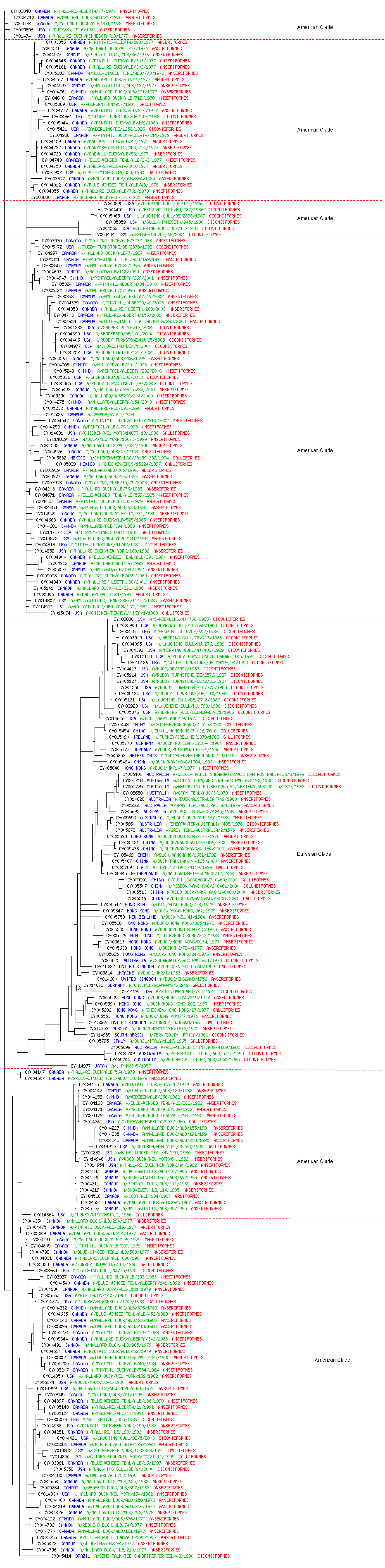
CY003848 CANADA A/MALLARD/ALBERTA/77/1977 ANSERIFORMES
CY004710 CANADA A/MALLARD DUCK/ALB/26/1976 ANSERIFORMES
CY004784 CANADA A/MALLARD DUCK/ALB/354/1978 ANSERIFORMES
CY005896 USA A/DUCK/MN/1516/1981 ANSERIFORMES
CY014740 USA A/MALLARD DUCK/MINNESOTA/19/1979 ANSERIFORMES
American Clade
CY003856 CANADA A/PINTAIL/ALBERTA/293/1977 ANSERIFORMES
CY004318 CANADA A/MALLARD DUCK/ALB/57/1976 ANSERIFORMES
CY004577 CANADA A/PINTAIL DUCK/ALB/86/1976 ANSERIFORMES
CY004346 CANADA A/PINTAIL DUCK/ALB/303/1977 ANSERIFORMES
CY005181 CANADA A/MALLARD DUCK/ALB/302/1977 ANSERIFORMES
CY005188 CANADA A/BLUE-WINGED TEAL/ALB/778/1978 ANSERIFORMES
CY004467 CANADA A/MALLARD DUCK/ALB/46/1977 ANSERIFORMES
CY004593 CANADA A/MALLARD DUCK/ALB/127/1977 ANSERIFORMES
CY004961 CANADA A/MALLARD DUCK/ALB/291/1977 ANSERIFORMES
CY004600 CANADA A/MALLARD DUCK/ALB/712/1978 ANSERIFORMES
CY005889 USA A/PHEASANT/MN/917/1980 GALLIFORMES
CY004777 CANADA A/PINTAIL DUCK/ALB/220/1977 ANSERIFORMES
CY004861 USA A/RUDDY TURNSTONE/DE/512/1988 CICONIIFORMES
CY005044 CANADA A/PINTAIL DUCK/ALB/169/1980 ANSERIFORMES
CY005421 USA A/SANDERLING/DE/1258/1986 CICONIIFORMES
CY004988 CANADA A/PINTAIL DUCK/ALBERTA/114/1979 ANSERIFORMES
CY004459 CANADA A/MALLARD DUCK/ALB/42/1977 ANSERIFORMES
CY004722 CANADA A/CANVASBACK DUCK/ALB/274/1977 ANSERIFORMES
CY004729 CANADA A/GADWALL DUCK/ALB/53/1977 ANSERIFORMES
CY004743 CANADA A/BLUE-WINGED TEAL/ALB/243/1977 ANSERIFORMES
CY004750 CANADA A/MALLARD/ALBERTA/300/1977 ANSERIFORMES
CY005907 USA A/TURKEY/MINNESOTA/833/1980 GALLIFORMES
CY003872 CANADA A/MALLARD DUCK/ALB/884/1984 ANSERIFORMES
CY004012 CANADA A/BLUE-WINGED TEAL/ALB/48/1978 ANSERIFORMES
CY004055 CANADA A/MALLARD DUCK/ALB/761/1978 ANSERIFORMES
CY003880 CANADA A/MALLARD DUCK/ALB/376/1985 ANSERIFORMES
CY003895 USA A/HERRING GULL/DE/475/1986 CICONIIFORMES
CY004451 USA A/HERRING GULL/NJ/782/1986 CICONIIFORMES
CY005065 USA A/LAUGHING GULL/DE/2838/1987 CICONIIFORMES
CY005859 USA A/GULL/MINNESOTA/945/1980 CICONIIFORMES
CY004562 USA A/HERRING GULL/DE/712/1988 CICONIIFORMES
CY004444 USA A/SHOREBIRD/DE/68/2004 CICONIIFORMES
American Clade
CY003930 CANADA A/MALLARD DUCK/ALB/323/1988 ANSERIFORMES
CY005072 USA A/RUDDY TURNSTONE/DE/2378/1988 CICONIIFORMES
CY004997 CANADA A/MALLARD DUCK/ALB/7/1987 ANSERIFORMES
CY005351 CANADA A/GREEN-WINGED TEAL/ALB/199/1991 ANSERIFORMES
CY003953 CANADA A/MALLARD/ALB/202/1996 ANSERIFORMES
CY004693 CANADA A/MALLARD/ALB/118/1995 ANSERIFORMES
CY004947 CANADA A/PINTAIL/ALBERTA/269/2001 ANSERIFORMES
CY005324 CANADA A/PINTAIL/ALBERTA/84/2000 ANSERIFORMES
CY005225 CANADA A/MALLARD/ALB/5/1995 ANSERIFORMES
CY003985 CANADA A/MALLARD/ALBERTA/149/2002 ANSERIFORMES
CY004339 CANADA A/PINTAIL/ALBERTA/49/2003 ANSERIFORMES
CY004353 CANADA A/MALLARD/ALBERTA/209/2003 ANSERIFORMES
CY004703 CANADA A/MALLARD/ALBERTA/156/2001 ANSERIFORMES
CY004954 CANADA A/BLUE-WINGED TEAL/ALBERTA/293/2003 ANSERIFORMES
CY004283 USA A/SHOREBIRD/DE/12/2004 CICONIIFORMES
CY004399 USA A/SHOREBIRD/DE/101/2004 CICONIIFORMES
CY004406 USA A/RUDDY TURNSTONE/NJ/65/1985 CICONIIFORMES
CY004977 USA A/SHOREBIRD/DE/75/2004 CICONIIFORMES
CY005257 USA A/SHOREBIRD/DE/122/2004 CICONIIFORMES
CY004267 CANADA A/MALLARD/ALB/206/1996 ANSERIFORMES
CY004508 CANADA A/MALLARD/ALB/201/1998 ANSERIFORMES
CY005243 CANADA A/PINTAIL/ALBERTA/202/2000 ANSERIFORMES
CY005331 USA A/SHOREBIRD/DE/236/2003 CICONIIFORMES
CY005365 USA A/RUDDY TURNSTONE/DE/97/2000 CICONIIFORMES
CY005093 CANADA A/MALLARD/ALBERTA/34/2001 ANSERIFORMES
CY005250 CANADA A/MALLARD/ALBERTA/208/2000 ANSERIFORMES
CY004275 CANADA A/MALLARD/ALBERTA/154/2003 ANSERIFORMES
CY005232 CANADA A/MALLARD/ALB/196/1996 ANSERIFORMES
CY015007 CANADA A/CANADA/RV504/2004
CY004547 CANADA A/PINTAIL DUCK/ALBERTA/210/2002 ANSERIFORMES
CY004259 CANADA A/PINTAIL/ALB/179/1993 ANSERIFORMES
CY014881 USA A/CHICKEN/NEW YORK/14677-13/1998 GALLIFORMES
CY014889 USA A/DUCK/NEW YORK/16873/1999 ANSERIFORMES
CY004532 CANADA A/MALLARD DUCK/ALB/322/1988 ANSERIFORMES
CY004926 CANADA A/MALLARD/ALB/47/1998 ANSERIFORMES
CY005832 MEXICO A/CHICKEN/HIDALGO/28159-232/1994 GALLIFORMES
CY005839 MEXICO A/CHICKEN/CHIS/15224/1997 GALLIFORMES
American Clade
CY003969 CANADA A/MALLARD/ALB/205/1998 ANSERIFORMES
CY003977 CANADA A/MALLARD/ALB/226/1998 ANSERIFORMES
CY003993 CANADA A/MALLARD/ALBERTA/79/2003 ANSERIFORMES
CY004203 CANADA A/MALLARD DUCK/ALB/76/1985 ANSERIFORMES
CY004671 CANADA A/BLUE-WINGED TEAL/ALB/569/1985 ANSERIFORMES
CY004483 CANADA A/PINTAIL DUCK/ALB/238/1979 ANSERIFORMES
CY004854 CANADA A/PINTAIL DUCK/ALB/623/1985 ANSERIFORMES
CY014549 CANADA A/MALLARD DUCK/ALBERTA/331/1985 ANSERIFORMES
CY004663 CANADA A/MALLARD DUCK/ALB/525/1985 ANSERIFORMES
CY004681 CANADA A/MALLARD/ALB/394/1988 ANSERIFORMES
CY014787 USA A/TURKEY/MINNESOTA/1/1988 GALLIFORMES
CY014873 USA A/BLACK DUCK/NEW YORK/184/1988 ANSERIFORMES
CY004818 USA A/RUDDY TURNSTONE/NJ/47/1985 CICONIIFORMES
CY014858 USA A/MALLARD DUCK/NEW YORK/180/1986 ANSERIFORMES
CY004904 CANADA A/BLUE-WINGED TEAL/ALB/293/1994 ANSERIFORMES
CY004912 CANADA A/MALLARD/ALB/49/1995 ANSERIFORMES
CY005002 CANADA A/MALLARD/ALB/194/1992 ANSERIFORMES
CY005058 CANADA A/MALLARD DUCK/ALB/435/1985 ANSERIFORMES
CY004940 CANADA A/MALLARD/ALBERTA/30/2001 ANSERIFORMES
CY005141 CANADA A/MALLARD DUCK/ALB/321/1988 ANSERIFORMES
CY005305 CANADA A/MALLARD/ALB/124/1991 ANSERIFORMES
CY014807 USA A/MALLARD DUCK/TENNESSEE/11457/1985 ANSERIFORMES
CY014902 USA A/MALLARD DUCK/NEW YORK/170/1982 ANSERIFORMES
CY015074 USA A/CHICKEN/PENNSYLVANIA/1/1983 GALLIFORMES
CY003888 USA A/SANDERLING/NJ/766/1986 CICONIIFORMES
CY003908 USA A/HERRING GULL/DE/698/1988 CICONIIFORMES
CY004555 USA A/HERRING GULL/DE/692/1988 CICONIIFORMES
CY003915 USA A/HERRING GULL/DE/703/1988 CICONIIFORMES
CY004095 USA A/LAUGHING GULL/NJ/276/1989 CICONIIFORMES
CY004392 USA A/HERRING GULL/NJ/402/1989 CICONIIFORMES
CY015128 USA A/RUDDY TURNSTONE/DELAWARE/105/1998 CICONIIFORMES
CY015136 USA A/RUDDY TURNSTONE/DELAWARE/34/1993 CICONIIFORMES
CY004413 USA A/KNOT/DE/2552/1987 CICONIIFORMES
CY005114 USA A/RUDDY TURNSTONE/DE/2576/1987 CICONIIFORMES
CY005127 USA A/RUDDY TURNSTONE/DE/2731/1987 CICONIIFORMES
CY004568 USA A/RUDDY TURNSTONE/DE/773/1988 CICONIIFORMES
CY005134 USA A/RUDDY TURNSTONE/DE/510/1988 CICONIIFORMES
CY005121 USA A/LAUGHING GULL/DE/2718/1987 CICONIIFORMES
CY003923 USA A/LAUGHING GULL/NJ/798/1986 CICONIIFORMES
CY005376 USA A/LAUGHING GULL/DELAWARE/471/1986 CICONIIFORMES
1
2
CY014646 USA A/GULL/MARYLAND/19/1977 CICONIIFORMES
CY005448 CHINA A/CHICKEN/NANCHANG/7-010/2000 GALLIFORMES
CY005454 CHINA A/QUAIL/NANCHANG/7-026/2000 GALLIFORMES
CY015090 IRELAND A/TURKEY/IRELAND/1378/1983 GALLIFORMES
CY005770 GERMANY A/DUCK/POTSDAM/2216-4/1984 ANSERIFORMES
CY005777 GERMANY A/DUCK/POTSDAM/1402-6/1986 ANSERIFORMES
CY005852 NETHERLANDS A/SHOVELER/NETHERLANDS/19/1999 ANSERIFORMES
CY005494 CHINA A/DUCK/NANCHANG/1904/1992 ANSERIFORMES
CY005640 HONG KONG A/DUCK/HK/167/1977 ANSERIFORMES
CY005406 AUSTRALIA A/WEDGE-TAILED SHEARWATER/WESTERN AUSTRALIA/2576/1979 CICONIIFORMES
CY005718 AUSTRALIA A/SOOTY TERN/WESTERN AUSTRALIA/2190/1983 CICONIIFORMES
CY005725 AUSTRALIA A/WEDGE-TAILED SHEARWATER/WESTERN AUSTRALIA/2327/1983 CICONIIFORMES
CY005680 AUSTRALIA A/GRAY TEAL/AUS/3/1979 ANSERIFORMES
CY014628 AUSTRALIA A/DUCK/AUSTRALIA/749/1980 ANSERIFORMES
CY005666 AUSTRALIA A/GRAY TEAL/AUSTRALIA/1/1979 ANSERIFORMES
CY005692 AUSTRALIA A/BLACK DUCK/AUS/4045/1980 ANSERIFORMES
CY005653 AUSTRALIA A/BLACK DUCK/AUS/751/1978 ANSERIFORMES
CY005660 AUSTRALIA A/SHEARWATER/AUSTRALIA/405/1978 CICONIIFORMES
CY005673 AUSTRALIA A/GREY TEAL/AUSTRALIA/2/1979 ANSERIFORMES
CY005598 HONG KONG A/DUCK/HONG KONG/073/1976 ANSERIFORMES
CY005431 CHINA A/DUCK/NANCHANG/2-0486/2000 ANSERIFORMES
CY005438 CHINA A/DUCK/NANCHANG/4-184/2000 ANSERIFORMES
CY005469 CHINA A/DUCK/NANCHANG/1681/1992 ANSERIFORMES
CY005487 CHINA A/DUCK/NANCHANG/4-165/2000 ANSERIFORMES
CY005789 ITALY A/TURKEY/ITALY/4169/1999 GALLIFORMES
CY005845 NETHERLANDS A/MALLARD/NETHERLANDS/12/2000 ANSERIFORMES
Eurasian Clade
CY005501 CHINA A/QUAIL/NANCHANG/2-0460/2000 GALLIFORMES
CY005507 CHINA A/PIGEON/NANCHANG/2-0461/2000 COLUMBIFORMES
CY005513 CHINA A/WILD DUCK/NANCHANG/2-0480/2000 ANSERIFORMES
CY005519 CHINA A/CHICKEN/NANCHANG/4-301/2001 GALLIFORMES
CY005547 HONG KONG A/DUCK/HONG KONG/278/1978 ANSERIFORMES
CY005647 HONG KONG A/DUCK/HONG KONG/562/1979 ANSERIFORMES
CY005759 NEW ZEALAND A/DUCK/NZL/41/1984 ANSERIFORMES
CY005568 HONG KONG A/DUCK/HONG KONG/365/1978 ANSERIFORMES
CY005583 HONG KONG A/GOOSE/HONG KONG/23/1978 ANSERIFORMES
CY005576 HONG KONG A/DUCK/HONG KONG/342/1978 ANSERIFORMES
CY005613 HONG KONG A/DUCK/HONG KONG/D134/1977 ANSERIFORMES
CY005633 HONG KONG A/DUCK/HK/784/1979 ANSERIFORMES
CY005625 HONG KONG A/DUCK/HONG KONG/24/1976 ANSERIFORMES
CY005823 AUSTRALIA A/SHEARWATER/AUSTRALIA/1/1973 CICONIIFORMES
CY015082 UNITED KINGDOM A/CHICKEN/SCOTLAND/1959 GALLIFORMES
CY005814 UKRAINE A/DUCK/UKR/1/1963 ANSERIFORMES
CY014680 UNITED KINGDOM A/DUCK/ENGLAND/1956 ANSERIFORMES
CY014672 GERMANY A/CHICKEN/GERMANY/N/1949 GALLIFORMES
3
CY014695 USA A/GULL/MARYLAND/704/1977 CICONIIFORMES
CY005539 HONG KONG A/DUCK/HONG KONG/319/1978 ANSERIFORMES
CY005590 HONG KONG A/GULL/HONG KONG/205/1977 CICONIIFORMES
CY005606 HONG KONG A/CHICKEN/HONG KONG/17/1977 GALLIFORMES
CY005553 HONG KONG A/DUCK/HONG KONG/7/1975 ANSERIFORMES
CY015066 UNITED KINGDOM A/TURKEY/ENGLAND/1963 GALLIFORMES
CY014703 RUSSIA A/DUCK/CHABAROVSK/1610/1972 ANSERIFORMES
CY014985 SOUTH AFRICA A/TERN/SOUTH AFRICA/1961 CICONIIFORMES
CY005795 ITALY A/GULL/ITALY/1117/1965 CICONIIFORMES
CY005699 AUSTRALIA A/RED-NECKED STINT/AUS/4189/1980 CICONIIFORMES
CY005709 AUSTRALIA A/RED-NECKED STINT/AUS/5745/1981 CICONIIFORMES
CY005704 AUSTRALIA A/RED-NECKED STINT/AUS/4500/1980 CICONIIFORMES
CY014977 JAPAN A/JAPAN/305/1957
CY004107 CANADA A/MALLARD DUCK/ALB/564/1979 ANSERIFORMES
CY004607 CANADA A/GREEN-WINGED TEAL/ALB/430/1979 ANSERIFORMES
CY004115 CANADA A/PINTAIL DUCK/ALB/628/1979 ANSERIFORMES
CY004147 CANADA A/PINTAIL DUCK/ALB/189/1982 ANSERIFORMES
CY004155 CANADA A/WIDGEON/ALB/256/1982 ANSERIFORMES
CY004163 CANADA A/BLUE-WINGED TEAL/ALB/266/1982 ANSERIFORMES
CY004171 CANADA A/MALLARD DUCK/ALB/289/1982 ANSERIFORMES
CY004179 CANADA A/BLUE-WINGED TEAL/ALB/685/1982 ANSERIFORMES
CY014765 USA A/TURKEY/MINNESOTA/957/1980 GALLIFORMES
CY004227 CANADA A/MALLARD DUCK/ALB/155/1990 ANSERIFORMES
CY004235 CANADA A/MALLARD DUCK/ALB/191/1990 ANSERIFORMES
CY004243 CANADA A/MALLARD DUCK/ALB/253/1990 ANSERIFORMES
CY014910 USA A/CHICKEN/NEW YORK/28263/1989 GALLIFORMES
CY005882 USA A/BLUE-WINGED TEAL/MN/993/1980 ANSERIFORMES
CY014946 USA A/WOOD DUCK/NEW YORK/66/1982 ANSERIFORMES
CY014954 USA A/MALLARD DUCK/NEW YORK/90/1982 ANSERIFORMES
American Clade
CY004187 CANADA A/MALLARD DUCK/ALB/10/1985 ANSERIFORMES
CY004195 CANADA A/BLUE-WINGED TEAL/ALB/69/1985 ANSERIFORMES
CY004211 CANADA A/PINTAIL DUCK/ALB/111/1985 ANSERIFORMES
CY004219 CANADA A/SHOVELER/ALB/114/1985 ANSERIFORMES
CY004516 CANADA A/COOT/ALB/134/1987 GRUIFORMES
CY004524 CANADA A/MALLARD DUCK/ALB/294/1987 ANSERIFORMES
CY005107 CANADA A/MALLARD DUCK/ALB/98/1985 ANSERIFORMES
CY014664 USA A/TURKEY/WISCONSIN/1/1966 GALLIFORMES
CY004360 CANADA A/MALLARD DUCK/ALB/294/1977 ANSERIFORMES
CY004475 CANADA A/PINTAIL DUCK/ALB/219/1977 ANSERIFORMES
CY005009 CANADA A/MALLARD DUCK/ALB/224/1977 ANSERIFORMES
CY004791 CANADA A/MALLARD DUCK/ALB/106/1979 ANSERIFORMES
CY004805 CANADA A/PINTAIL DUCK/ALB/599/1979 ANSERIFORMES
CY004798 CANADA A/BLUE-WINGED TEAL/ALB/580/1979 ANSERIFORMES
CY004831 CANADA A/MALLARD DUCK/ALB/630/1984 ANSERIFORMES
CY005828 CANADA A/TURKEY/ONTARIO/6118/1968 GALLIFORMES
CY003864 USA A/LAUGHING GULL/NJ/75/1985 CICONIIFORMES
CY003937 CANADA A/MALLARD DUCK/ALB/353/1988 ANSERIFORMES
CY004540 CANADA A/BLUE-WINGED TEAL/ALBERTA/141/1992 ANSERIFORMES
CY004130 CANADA A/MALLARD DUCK/ALB/1151/1979 ANSERIFORMES
CY005867 USA A/PIGEON/MN/1407/1981 COLUMBIFORMES
CY014779 USA A/TURKEY/MINNESOTA/1200/1980 GALLIFORMES
CY004332 CANADA A/MALLARD DUCK/ALB/396/1983 ANSERIFORMES
CY004635 CANADA A/BLUE-WINGED TEAL/ALB/452/1983 ANSERIFORMES
CY004643 CANADA A/MALLARD DUCK/ALB/506/1983 ANSERIFORMES
CY005099 CANADA A/MALLARD DUCK/ALB/743/1983 ANSERIFORMES
CY005274 CANADA A/MALLARD DUCK/ALB/797/1983 ANSERIFORMES
CY005344 CANADA A/MALLARD DUCK/ALBERTA/342/1983 ANSERIFORMES
CY004491 CANADA A/MALLARD DUCK/ALB/965/1979 ANSERIFORMES
CY004614 CANADA A/PINTAIL DUCK/ALB/462/1979 ANSERIFORMES
CY005051 CANADA A/GREEN-WINGED TEAL/ALB/228/1985 ANSERIFORMES
American Clade
CY005200 CANADA A/MALLARD DUCK/ALB/40/1984 ANSERIFORMES
CY005207 CANADA A/PINTAIL DUCK/ALB/584/1984 ANSERIFORMES
CY014850 USA A/MALLARD DUCK/NEW YORK/189/1982 ANSERIFORMES
CY005874 USA A/GOOSE/MN/5733-1/1980 ANSERIFORMES
CY014969 USA A/MALLARD DUCK/NEW YORK/6861/1978 ANSERIFORMES
CY003945 CANADA A/MALLARD/ALB/201/1996 ANSERIFORMES
CY004897 CANADA A/BLUE-WINGED TEAL/ALB/136/1990 ANSERIFORMES
CY005148 CANADA A/MALLARD/ALBERTA/11/1991 ANSERIFORMES
CY005154 CANADA A/MALLARD/ALB/17/1991 ANSERIFORMES
CY005079 USA A/RED KNOT/NJ/325/1989 CICONIIFORMES
CY014938 USA A/PINTAIL DUCK/NEW YORK/155/1982 ANSERIFORMES
CY004251 CANADA A/MALLARD/ALB/199/1992 ANSERIFORMES
CY004421 USA A/LAUGHING GULL/DE/5/2003 CICONIIFORMES
CY005086 CANADA A/PINTAIL/ALBERTA/129/1993 ANSERIFORMES
CY014822 USA A/CHICKEN/NEW YORK/13828-3/1995 GALLIFORMES
CY014830 USA A/GUINEA FOWL/NEW YORK/20221-11/1995 GALLIFORMES
CY003961 CANADA A/BLUE-WINGED TEAL/ALB/16/1997 ANSERIFORMES
CY005358 USA A/LAUGHING GULL/DE/94/2000 CICONIIFORMES
CY004380 CANADA A/MALLARD/ALB/52/1997 ANSERIFORMES
CY004650 CANADA A/MALLARD DUCK/ALB/635/1983 ANSERIFORMES
CY005264 CANADA A/REDHEAD DUCK/ALB/357/1983 ANSERIFORMES
CY014930 USA A/MALLARD DUCK/NEW YORK/194/1982 ANSERIFORMES
CY004000 CANADA A/MALLARD DUCK/ALB/250/1978 ANSERIFORMES
CY004019 CANADA A/MALLARD DUCK/ALB/280/1978 ANSERIFORMES
CY004026 CANADA A/MALLARD DUCK/ALB/290/1978 ANSERIFORMES
CY004122 CANADA A/MALLARD DUCK/ALB/676/1979 ANSERIFORMES
CY004736 CANADA A/REDHEAD DUCK/ALB/74/1977 ANSERIFORMES
CY004770 CANADA A/MALLARD DUCK/ALB/161/1977 ANSERIFORMES
CY005016 CANADA A/BLUE-WINGED TEAL/ALB/295/1977 ANSERIFORMES
CY005023 CANADA A/WIDGEON/ALB/284/1977 ANSERIFORMES
CY004756 CANADA A/MALLARD DUCK/ALB/210/1977 ANSERIFORMES
CY005414 BRAZIL A/SEMI-PALMATED SANDPIPER/BRAZIL/43/1990 CICONIIFORMES
